# ISD3: a particokinetic model for predicting the combined effects of particle sedimentation, diffusion and dissolution on cellular dosimetry for in vitro systems

**DOI:** 10.1186/s12989-018-0243-7

**Published:** 2018-01-25

**Authors:** Dennis G. Thomas, Jordan N. Smith, Brian D. Thrall, Donald R. Baer, Hadley Jolley, Prabhakaran Munusamy, Vamsi Kodali, Philip Demokritou, Joel Cohen, Justin G. Teeguarden

**Affiliations:** 1 Computational Biology, Biological Sciences Division, Pacific Northwest National Laboratory, 902 Battelle Blvd, Richland, WA 99352 USA; 2 Health Effects and Exposure Science, Biological Sciences Division, Pacific Northwest National Laboratory, 902 Battelle Blvd, Richland, WA 99352 USA; 3 Interfacial Sciences and Simulation, Environmental Molecular Sciences Division, Pacific Northwest National Laboratory, Richland, WA 99352 USA; 4 Center for Nanotechnology and Nanotoxicology, Department of Environmental Health, Harvard University T. H. Chan School of Public Health, Boston, MA 02115 USA; 5 Department of Environmental and Molecular Toxicology, Oregon State University, Corvallis, OR 93771 USA

**Keywords:** Nanoparticle, Dissolution, Population balance equation, Nanosilver, In vitro dosimetry, Particokinetic model, ISDD, ISD3

## Abstract

**Background:**

The development of particokinetic models describing the delivery of insoluble or poorly soluble nanoparticles to cells in liquid cell culture systems has improved the basis for dose-response analysis, hazard ranking from high-throughput systems, and now allows for translation of exposures across in vitro and in vivo test systems. Complimentary particokinetic models that address processes controlling delivery of both particles and released ions to cells, and the influence of particle size changes from dissolution on particle delivery for cell-culture systems would help advance our understanding of the role of particles and ion dosimetry on cellular toxicology. We developed ISD3, an extension of our previously published model for insoluble particles, by deriving a specific formulation of the Population Balance Equation for soluble particles.

**Results:**

ISD3 describes the time, concentration and particle size dependent dissolution of particles, their delivery to cells, and the delivery and uptake of ions to cells in in vitro liquid test systems. We applied the model to calculate the particle and ion dosimetry of nanosilver and silver ions in vitro after calibration of two empirical models, one for particle dissolution and one for ion uptake. Total media ion concentration, particle concentration and total cell-associated silver time-courses were well described by the model, across 2 concentrations of 20 and 110 nm particles. ISD3 was calibrated to dissolution data for 20 nm particles as a function of serum protein concentration, but successfully described the media and cell dosimetry time-course for both particles at all concentrations and time points. We also report the finding that protein content in media affects the initial rate of dissolution and the resulting near-steady state ion concentration in solution for the systems we have studied.

**Conclusions:**

By combining experiments and modeling, we were able to quantify the influence of proteins on silver particle solubility, determine the relative amounts of silver ions and particles in exposed cells, and demonstrate the influence of particle size changes resulting from dissolution on particle delivery to cells in culture. ISD3 is modular and can be adapted to new applications by replacing descriptions of dissolution, sedimentation and boundary conditions with those appropriate for particles other than silver.

**Electronic supplementary material:**

The online version of this article (10.1186/s12989-018-0243-7) contains supplementary material, which is available to authorized users.

## Background

The biological effects of nanoparticles occur primarily at the cellular level, through interactions with structural and functional cell components [1, 2]. These cellular targets—membranes, membrane receptors, organelles like the lysosome, are present at sites of, for example cells within portal of entry tissues such as the lung [3], and distal to the portal of entry in systemic tissues like the liver [4], kidney [5], spleen [6] and the cardiovascular system [7]. The use of target-site exposure, or dosimetry, rather than less specific measures exposure such as nominal concentration or administered dose, has been shown to improve correlations between dose and response for drugs, chemicals, and inhaled gases and particles [8–12]. Increasingly, target site (e.g. tissue or cell) exposure has become a preferred metric for dose-response assessment in nanotoxicology [13–22].

The importance of cellular dosimetry of nanomaterials for in vitro toxicity studies is well established [13–24]. The rate and extent of particle delivery to cells residing in a liquid test system is determined by features of the material—size, density, agglomeration state, agglomerate density—and the system, for example media density, viscosity and height [25]. These factors have been shown to produce significant differences in the target cell dose in in vitro systems for equivalent nominal media concentrations of multiple particles. These differences can lead directly to errors in the relative or absolute potency (hazard) of assayed particles, particularly when particle differences that affect diffusion and sedimentation are large [13, 14, 16, 25]. To address this issue, computational models [14, 15, 26] and experimental methods for dosimetry [18, 22] have been developed and applied to these test systems.

Hinderliter et al. [14] developed a computational model called ISDD (in vitro sedimentation, diffusion and target cell dosimetry), to compute the cell-associated dose fraction of nanoparticles administered to cells in in vitro toxicity studies. This model accounts for the deposition of particles onto the cells, due to particle sedimentation and diffusion in the liquid column above the cells. The ISDD model, however, cannot be applied to nanoparticles that change in size due to dissolution while they diffuse and settle down the liquid column. For example, the in vitro cytotoxicity of silver nanoparticles (AgNPs) is induced by the silver ions that get released from the surface of the particles due to dissolution in cell culture media [27]. Therefore, to correctly interpret the cytotoxicity data of silver nanoparticles, it becomes important to quantify not only the amount of particles associated with the cells but also the amount of ions. While the dissolution process reduces the size of the particles in the liquid medium, the cellular uptake of particles that reach the cell surface (via diffusion and sedimentation) reduces the particle number in the medium. When particles change size, the rates at which they diffuse, sediment (in the liquid media) and dissolve will also change. The net effect is a temporal change in the size distribution and number of particles at every location along the height of the liquid column. Consequently, the mass, surface area, and volume of particles in the liquid media and in the cells will be temporally different depending on the temporal size distribution. Therefore, it becomes necessary to simultaneously track the temporal changes in both the number and size of the particles in the liquid medium. Moreover, the rate of dissolution depends on the surface area of all the particles in the liquid medium and the concentration of the dissolved solute (ions). Hence, if there are particles of various sizes, then it will be necessary to simultaneously track the temporal changes of all particle size classes. For such a case, it is not logical to run independent simulations for each size class of particles and then calculate the dose fraction of particles deposited in the cells, as was done previously with the ISDD model [14].

To account for dissolution effects, DeLoid et al. [15] developed the one-dimensional Distorted Grid (DG) model, which also considers the simultaneous tracking of the size distribution of polydisperse particles. The dissolution kinetics was however not modeled as a particle surface area limited mechanism, nor was it validated against any experimental data. Instead, the dissolution was simply modeled as a reduction in agglomerate size in proportion to the extent of dissolution. Although the DG model was validated for insoluble industrially relevant nanoparticles (TiO_2_, SiO_2_) suspended in commonly used culture media by computational fluid dynamics modeling and analysis of frozen sections along the liquid column over time, it was not experimentally validated for soluble particles. Specifically, the DG model was applied to ZnO agglomerate nanoparticles under different scenarios of dissolved ZnO concentrations and dissolution rates. Realistic scenarios should include the effect of proteins in the media [28] and the reduction in size proportional to the particle surface area. On the other hand, a more comprehensive approach was followed in the Agglomeration-diffusion-sedimentation-reaction model (ADSRM), developed by Mukherjee et al. [26]. This model accounted for both particle agglomeration and surface area limited dissolution during transport, and was applied specifically to citrate-coated silver nanoparticles. Although the model incorporated detailed mechanisms of silver oxidation and citrate reduction reactions, it was not applied for the relevant media nor did it consider protein effects - the media used was acid solutions of different pH values (3, 5, and 7). Therefore, it is also not clear whether dynamic agglomeration is important or not for silver nanoparticles under realistic media conditions. Moreover, the comparisons between the model predictions of AgNP dissolution and their in vitro measurements over 14 days (shown in Figure 9 of their paper) clearly indicate that the model does not capture the initial high and later slow rates of AgNP dissolution.

Here, we extend our work on the ISDD model to incorporate dissolution effects for *soluble* nanoparticles under realistic media conditions. Consequently, we have developed a new in vitro dosimetry model, called ISD3 – the **i**n vitro **s**edimentation, **d**iffusion, **d**issolution, and **d**osimetry model. This model combines the effect of particle dissolution kinetics with effects of sedimentation and diffusion, to compute the amount of particles and ions delivered to cells. The model accounts for simultaneous changes in both the number and size of particles in the liquid media, by solving for the number density of particles as a function of size and spatial location, based on a population balance formalism [29–31]. The effect of dynamic agglomeration of particles is not considered because it was not found relevant for the test system under study, although it can easily be incorporated within the population balance framework. ISD3 is modular, allowing adaptation by inclusion of alternative boundary conditions, models of uptake, dissolution, or sedimentation of agglomerates. The model is described below, followed by results from a validation study of the ISD3 approach (of incorporating dissolution effects) based on the transport and dissolution properties of silver nanoparticles (20 and 110 nm) in 10% fetal bovine serum (FBS) solution.

## Methods

### Experimental methods

#### Chemicals

RPMI 1640 Medium was obtained from Gibco Life Technologies (Grand Island, NY, USA). Fetal bovine serum (FBS) was purchased from Atlanta Biologicals (Flowery Branch, GA, USA). Concentrated double-distilled nitric and hydrochloric acids were obtained from GFS Chemicals, Inc. (Columbus, OH, USA). Certified silver standard was acquired from VHG Labs, Inc. (Manchester, NH, USA). Silver acetate (99.99%) and other general laboratory chemicals were acquired from Sigma-Aldrich (St. Louis, MO, USA).

#### Nanoparticles

Citrate-coated silver particles with primary diameters of 20 and 110 nm containing a gold core of 7 nm manufactured by nanoComosix (San Diego, CA, USA) at a concentration of 1 mg/mL were provided by the National Institute of Environmental Health Sciences (NIEHS) Centers for Nanotechnology Health Implications Research (NCNHIR). These particles were reported to have hydrodynamic diameters of 24 and 104 nm, respectively, in water by the Nanotechnology Characterization Laboratory (NCL) using Dynamic Light Scattering (DLS) with a Malvern Zetasizer Nano ZS instrument (Southborough, MA, USA) and core diameters of 20.3 and 111.5 nm by Transmission Electron Microscopy (TEM).

Hydrodynamic diameters of silver nanoparticles in RPMI were measured using DLS with a ZetaPALS zeta potential and particle size analyzer (Brookhaven Instruments Corporation, Holtsville, NY, USA). Hydrodynamic diameter of nanoparticles was calculated from intensity weighted average translational diffusion coefficient using cumulant analysis on the autocorrelation function using vendor provided software. Stock suspensions of nanoparticles were tested for endotoxin levels using a Toxinsensor Chromogenic LAL kit (GenScript, Piscataway, NJ, USA). The concentration of nanoparticles was 100 μg/mL for DLS analysis.

The effective density of the nanoparticles was measured via the previously described volumetric centrifugation method (VCM) [22].

#### Nanoparticle dissolution

Dissolution of 20 and 110 nm silver nanoparticles was measured in RPMI cell culture media. An optimized dispersion protocol was used to consistently control nanoparticle agglomeration [32, 33]. Suspensions of silver nanoparticles (1 mL) were prepared in triplicate by mixing silver nanoparticle stock into FBS, followed by the addition of either RPMI cell culture media. This protocol enabled protein corona formation, preventing excessive agglomeration [32, 33]. Final concentrations of nanoparticle suspensions were 1–50 μg/mL with 1, 10, or 30% FBS. Nanoparticle suspensions were maintained in a cell culture incubator at standard conditions (37 C, ~ 5% CO_2_), and after incubating for 1–24 h, nanoparticle suspensions were removed and centrifuged at 30,000 rpm (49,000×g maximum, 38,000×g average, and 27,000×g minimum) for 90 min. After centrifugation, aliquots of supernatants (200 μL) were collected, and silver levels were quantified using inductively coupled plasma-mass spectrometry (ICP-MS).

#### Cellular uptake of nanoparticles and ions

Uptake of silver nanoparticles and ions were assessed in vitro. Rat alveolar macrophage cells (RAW 264.7) grown at standard cell culture conditions (37 C, ~ 5% CO_2_) were seeded in 6-well plates at 4 × 10^5^ and 2.5 × 10^5^ cells per well in RPMI 1640. Cell culture medium was supplemented with L-glutamine, Pen-Strep, and 10% FBS. Cells were incubated overnight and then dosed with 12.5 or 25 μg/mL 20 or 110 nm silver nanoparticle suspensions (3 mL) or 0.5 or 1.5 μg/mL silver ions from silver acetate. Nanoparticle dosing solutions were made by mixing silver nanoparticle stock into FBS, followed by the addition of cell culture media. After dosing, cells were incubated for 0.5–24 h. After incubation, cells were washed and scraped. A small aliquot (10 μL) was collected for cell counting using a hematocytometer. Total silver levels in remaining cells were quantified using ICP-MS.

#### Silver quantification

Silver levels in cell culture medium and cells were quantified using ICP-MS. Samples were spiked with ^89^Y as an internal standard and digested with 70% double distilled nitric acid (~ 2 mL) overnight until clear. Afterwards, double distilled concentrated hydrochloric acid (~ 1 mL) was added to shift the equilibrium from insoluble silver to soluble silver chloride complexes. Aliquots were diluted to 2% nitric acid, and total silver was quantified using an Agilent 7500 CE (Santa Clara, CA, USA) inductively coupled plasma-mass spectrometer. ^107^Ag measured in helium collision mode using ^45^Sc and ^115^In (10 ng/mL) as internal standards. Additionally, ^109^Ag was also monitored. Three rinses with 2% nitric acid between runs were used to minimize silver carryover. Quantification was accomplished using a linear regression fit to an external calibration curve. The calibration curve was made by spiking silver standards (VHG Labs, Inc., Manchester, NH, USA) in either cell culture medium or cells, depending on the sample matrix, and processed simultaneously with the samples. Limits of quantitation for silver were ~ 0.1 ng/mL for samples diluted to 2% nitric acid.

### ISD3 model overview

**Fig. 1:**
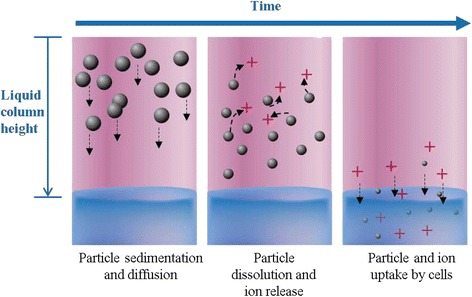
Processes represented in ISD3. Particles of different sizes settle and diffuse at different rates. Dissolution of particles reduces the size of particles and increases the concentration of ions in the liquid column. Dissolution and cellular uptake of particles reduce the number of particles for each particle size class. Ions are uniformly distributed in the liquid column, and their concentration increases due to dissolution and decreases due to cell uptake

Figure 1 depicts the various processes modeled in ISD3: 1) sedimentation and diffusion, 2) dissolution in the liquid media (e.g., in FBS); and, 3) cell uptake of ions and particles. The particles are treated as spherical in shape and can be modeled as primary particles, agglomerates, or as primary particles coated with proteins. Ions are modeled as a lumped system with a uniform concentration in the liquid media. The cellular uptake kinetics of ions is explicitly described, but the particles are assumed to be instantaneously taken up by the cells once they reach the cell surface (bottom surface of the liquid column). Dissolution of particles in the liquid media is considered; dissolution within cells is not.

The equations of the ISD3 model were developed in three steps. First, we used the population balance (PB) framework [30] to develop the equation for the particle number density, which characterized how the particles change in size and number in the liquid media as they undergo diffusion, sedimentation, and dissolution. Second, we performed experiments to develop and parameterize a kinetic model for describing the rate at which 20 and 110 nm silver particles dissolve in FBS containing cell culture liquid. Third, we performed experiments to develop and parameterize the kinetic model for characterizing the cellular uptake rate of the dissolved silver (ions). The kinetic models for the particle dissolution and cell ion-uptake were then integrated with sedimentation and diffusion models through the population balance framework, in order to capture the effects of dissolution on the deposition of silver particles in the liquid media.

The MATLAB code of the model is available for download at https://nanodose.pnnl.gov.

### Model derivation

#### Population balance equation for the particle number density

The population balance framework is a mathematical framework for describing the temporal behavior of a particulate system, where a population of particles (dispersed in a continuous environmental media) are continually created and/or destroyed by processes such as agglomeration, breakage, nucleation, and dissolution [30]. Generally, it seeks to describe the temporal behavior of the population of particles by solving for the number density via a series of state variables. State variable could be the position and any physical properties (e.g., diameter, surface area, volume) of the particle that can vary continuously due to the above processes, during transport [30].

In our system, we consider the liquid medium (above the cells) as a column of height *L* and uniform cross-sectional area *A* (volume, *V = L* × *A*). Assuming that no fluid convection, aggregation/agglomeration, coagulation and break-up of the particles occur in the liquid medium, and considering that net particle transport occurs only down the height of the liquid column (*x*), the equation for the particle number density function, *N*(*D*_*p*_; *x*, *t*) (units: #/length^2^) – the number of particles per unit area in the *x*-*D*_*p*_ parameter space – can be written as:

1 $$ \frac{\partial N\left({D}_p;x,t\right)}{\partial t}={D}_{diff}\left({D}_p\right)\frac{\partial^2N\left({D}_p;x,t\right)}{\partial {x}^2}-{V}_t\left({D}_p\right)\frac{\partial N\left({D}_p;x,t\right)}{\partial x}-\frac{\partial }{\partial {D}_p}\left(N\left({D}_p;x,t\right)\frac{\partial {D}_p}{\partial t}\right) $$

The diameter of the particles,*D*_*p*_, is the internal coordinate and the axial position, *x*, is the external coordinate over which the particle number density function *N*(*D*_*p*_; *x*, *t*) is defined. Therefore, the average number of particles in an infinitesimal volume of the coordinate space, *dD*_*p*_*dx*, is *N*(*D*_*p*_; *x*, *t*)*dD*_*p*_*dx*. Consequently, the total number of particles at any instant of time in the liquid medium is given by the relation,

2 $$ N(t)=\iint N\left({D}_p;x,t\right){dD}_p dx. $$

The three terms on the right-hand side of Eq. 1 describe how diffusion, sedimentation, and dissolution affect a net change in particle number density. These are described separately in the following sections.

##### Diffusion term

Fick’s law of diffusion is used to describe the rate at which particles of the same size diffuse down the liquid column. The isotropic diffusion coefficient,*D*_*diff*_ (in Eq. 1) for a spherical particle is defined as.

3 $$ {D}_{diff}=\frac{RT}{3{N}_A\pi \mu {D}_p}; $$

*R* is the universal gas constant (in units: J mol^− 1^ K^− 1^), *N*_*A*_ is the Avogadro’s number, *μ* is the dynamic viscosity (in units, N s m^− 2^), and *T* is the temperature of the liquid medium (units of K).

##### Sedimentation term

Stoke’s law is used to describe the rate at which particles of the same size sediment down the liquid column. Therefore, the sedimentation velocity,*V*_*t*_, of the particles (in Eq. 1) is defined by the Stoke’s relation:

4 $$ {V}_t=\frac{g\left({\rho}_p-{\rho}_f\right){D}_p^2}{18\mu }, $$

where *ρ*_*p*_ and *ρ*_*f*_are the density of the particles and the liquid media, respectively. If the particles are agglomerates, the equation for the sedimentation velocity (Eq. 4) can be replaced by the Sterling equation [34] to account for the fractal nature of the agglomerates; i.e.,

5 $$ {V}_t=\frac{g\left({\rho}_p-{\rho}_f\right){D}_p^{DF-1}{D}_{p1}^{3- DF}}{18\mu }. $$

In Eq. 5, *ρ*_*p*_ and *D*_*p*_refers to the density and diameter of the agglomerate (rather than that of the primary particles as in Eq. 4), and*D*_*p*1_ is primary particle diameter. The agglomerate density is calculated from the agglomerate porosity,*ε*, as

6 $$ {\rho}_p=\left(1-\varepsilon \right){\rho}_{p1}+{\varepsilon \rho}_f, $$

where *ρ*_*p*1_refers to primary particle density. The porosity is calculated using the equation

7 $$ \varepsilon =1-{\left(\frac{D_p}{D_{p1}}\right)}^{DF-3}, $$

where *DF* is the fractal dimension of the agglomerates. If the agglomerate density is known or measured, by the VCM, for example, then it is not calculated, but used as input parameter in Eqs. 6 and 7 to calculate the initial agglomerate porosity and diameter. If both the effective density and agglomerate diameters are known then they are directly used in Eq. 5. Alternative expressions that account for the permeability of the agglomerates can also be used [35, 36], as in the “particles in a box” sedimentation model [37], depending on the nature of the agglomerate state of the particles in the liquid media. In this work, we measured the density of silver particles using VCM, and determined that the particles were not agglomerates but protein-coated particles (discussed later).

##### Dissolution term

The dissolution term captures the rate at which the number of particles, of a given diameter (*D*_*p*_), changes as particles dissolve in the liquid media. The rate of decrease in the diameter of a particle is determined based on an experimentally characterized dissolution kinetic model.

#### A dissolution kinetic model for silver nanoparticles

We first applied the following two-parameter dissolution model to fit the 24-h dissolution data of the total dissolved ion concentration:

8 $$ \frac{dC^{diss,f}}{dt}=\frac{k_f\  Area(t)}{V}\ \left({C}_{sat}^{diss,f}-{C}^{diss,f,}\right); $$

where, *C*^*diss*, *f*^ is the free ion concentration (μg/mL) in the liquid medium, *k*_*f*_ is the solubility rate constant, ${C}_{sat}^{diss}$is the saturated ion concentration, *Area(t)* is the surface area of particles available for dissolution at any given time *t*, and *V* is the volume of the media. The model was not able to describe the biphasic, particle concentration dependent dissolution data. The formulation of the dissolution model was revised to include transfer to and from serum protein following experiments that revealed the influence of media protein on the rate and extent of dissolution.

The final model consists of two ordinary differential equations: one for the free ion concentration (*C*^*diss*, *f*^) and the other for the protein-bound ion concentration (*C*^*diss*, *p*^). The model for the free ion concentration is written as

9 $$ {\displaystyle \begin{array}{l}\frac{dC^{diss,f}}{dt}=\underset{\begin{array}{l}\mathrm{transfer}\  \mathrm{of}\  \mathrm{silver}\  \mathrm{ions}\  \mathrm{from}\  \mathrm{particles}\\ {}\mathrm{to}\  \mathrm{free}\kern0.5em \mathrm{ion}\ \mathrm{state}\end{array}}{\underbrace{\frac{k_f\  Area(t)}{V}\ \left({C}_{sat}^{diss,f}-{C}^{diss,f}\right)}}-\underset{\begin{array}{l}\mathrm{transfer}\  \mathrm{of}\  \mathrm{free}\kern0.5em \mathrm{silver}\  \mathrm{ions}\\ {}\mathrm{to}\  \mathrm{protein}\hbox{-} \mathrm{bound}\  \mathrm{state}\end{array}}{\underbrace{k_{f2p}{C}^{diss,f}\left(n\cdot {P}_0-{C}^{diss,p}\right)}}\\ {}\kern3.5em +\underset{\begin{array}{l}\mathrm{transfer}\  \mathrm{of}\  \mathrm{silver}\  \mathrm{ions}\  \mathrm{from}\\ {}\mathrm{protein}\hbox{-} \mathrm{bound}\  \mathrm{state}\  \mathrm{to}\  \mathrm{free}\\ {}\mathrm{ion}\ \mathrm{state}\end{array}}{\underbrace{k_{p2f}{C}^{diss,p}\left({C}_{sat}^{diss,f}-{C}^{diss,f}\right)}},\end{array}} $$

and the model for the protein-bound ion concentration is written as

10 $$ {\displaystyle \begin{array}{l}\frac{dC^{diss,p}}{dt}=\underset{\begin{array}{l}\mathrm{s}\mathrm{low}\  \mathrm{transfer}\  \mathrm{of}\  \mathrm{silver}\  \mathrm{ions}\  \mathrm{from}\\ {}\mathrm{particles}\  \mathrm{to}\  \mathrm{protein}\  \mathrm{bound}\  \mathrm{state}\end{array}}{\underbrace{\frac{k_p\  Area(t)}{V}\ \left(n\cdot {P}_0-{C}^{diss,p}\right)}}+\underset{\begin{array}{l}\mathrm{initial}\  \mathrm{fast}\  \mathrm{transfer}\  \mathrm{of}\  \mathrm{silver}\  \mathrm{ions}\  \mathrm{from}\ \\ {}\mathrm{particles}\  \mathrm{to}\  \mathrm{protein}\  \mathrm{bound}\  \mathrm{state}\end{array}}{\underbrace{\frac{k_{p2}\  Area(t)}{V}\ \left({n}_2\cdot {P}_0-{C}^{diss,p}\right)}}\\ {}+\underset{\begin{array}{l}\mathrm{binding}\  \mathrm{of}\  \mathrm{ions}\  \mathrm{from}\  \mathrm{solution}\\ {}\mathrm{to}\  \mathrm{protein}\mathrm{s}\end{array}}{\underbrace{k_{f2p}{C}^{diss,f}\left(n\cdot {P}_0-{C}^{diss,p}\right)}}-\underset{\begin{array}{l}\mathrm{transfer}\  \mathrm{of}\  \mathrm{silver}\  \mathrm{ions}\  \mathrm{from}\  \mathrm{protein}\hbox{-} \\ {}\mathrm{bound}\  \mathrm{state}\  \mathrm{to}\  \mathrm{free}\ \mathrm{ion}\ \mathrm{state}\end{array}}{\underbrace{k_{p2f}{C}^{diss,p}\left({C}_{sat}^{diss,f}-{C}^{diss,f}\right)}}.\\ {}\kern1.75em \end{array}} $$

Two different rates were included in the model for describing the transfer of ions from the particle surface to the proteins in order to capture the two rate behavior of the dissolution process (the first and second terms in Eq. 10).

There are eight fitted-parameters in the dissolution model equations (Eqs. 9 and 10): *k*_*f*_, the rate constant for the transfer of ions from the particle surface to the free ion state, in mL nm^− 2^ h^− 1^ units; ${C}_{sat}^{diss,f}$, the saturated concentration of free ions in solution, in μg mL^− 1^ units; *k*_*f2p*_, the rate constant for the transfer of free ions from solution to the protein-bound state, μg^− 1^ mL hr.^− 1^ units; *n* and *n*_*2*_ are the concentrations of ion binding sites available on the proteins per % of FBS, respectively, in μg mL^− 1^ FBS%^− 1^ units; *k*_*p2f*_, the rate constant for the transfer of ions from the protein-bound state to the free ion state, in units of μg^− 1^ mL hr.^− 1^; *k*_*p*_, the rate constant for the slow transfer of ions from the particle surface to the proteins, in units of mL nm^− 2^ h^− 1^; and, *k*_*p2*_, the rate constant for the initial fast transfer of silver ions from the particle surface to the protein-bound state, in mL nm^− 2^ h^− 1^ units. Parameter identifiability and sensitivity analyses were performed using functions (s*ensFun*, *colins*, and *sensRange*) from the FME [38] package in R [39]. A maximum of 6 parameters were identifiable based on the dissolution data of 20 nm particles in 1%, 10%, and 30% FBS concentrations (see Supporting Information).

### The cell uptake model for silver ions

The rate at which ions are taken by the cells is described as.

11 $$ \frac{dC^{diss, cell}}{dt}=\frac{D_{12}{SA}_2}{V}\frac{\left({C}^{diss,f}+{C}^{diss,p}-\frac{C^{diss, cell}V/{V}_{cell}}{PC_{21}}\right)}{Dis_2}; $$

*C*^*diss,cell*^is concentration of the ions in the cells (in μg/mL units, where mL refers to volume of the liquid media), *C*^*diss,f*^ is the concentration of free ions in the media (in μg/mL units), *C*^*diss,p*^ is the protein-bound ion concentration in the media (in μg/mL units), *V*_*cell*_ is the cell volume (in mL units), *t* is the time (in hr. units), *D*_12_is the diffusion coefficient (in cm^2^/h units), *SA*_*2*_ is the total cell surface area (in cm^2^ units), *Dis*_2_ is the thickness of the cell membrane (in cm units), and *PC*_21_ is the partition coefficient.

#### Final equations for the dissolved ion concentration

In addition to dissolution, the cell uptake of the dissolved ions will also determine the change in the concentration of the free and protein-bound ions (*C*^*diss,f*^ and *C*^*diss,p*^) in the liquid media. Based on Eq. 9 and the cellular uptake rate (Eq. 11), the final rate equation for the free ion concentration in the liquid media is,

12 $$ {\displaystyle \begin{array}{l}\frac{dC^{diss,f}}{dt}=\underset{\begin{array}{l}\mathrm{transfer}\  \mathrm{of}\  \mathrm{ions}\  \mathrm{from}\  \mathrm{particles}\\ {}\mathrm{to}\  \mathrm{free}\kern0.5em \mathrm{ion}\ \mathrm{state}\end{array}}{\underbrace{\frac{k_f\  Area(t)}{V}\ \left({C}_{sat}^{diss,f}-{C}^{diss,f}\right)}}-\underset{\begin{array}{l}\mathrm{tranfer}\  \mathrm{of}\  \mathrm{free}\  \mathrm{ions}\\ {}\mathrm{to}\  \mathrm{protein}\hbox{-} \mathrm{bound}\  \mathrm{state}\end{array}}{\underbrace{k_{f2p}{C}^{diss,f}\left(n\cdot {P}_0-{C}^{diss,p}\right)}}\\ {}\kern3.5em +\underset{\begin{array}{l}\mathrm{transfer}\  \mathrm{of}\  \mathrm{ions}\  \mathrm{from}\\ {}\mathrm{protein}\hbox{-} \mathrm{bound}\  \mathrm{state}\  \mathrm{to}\  \mathrm{free}\\ {}\mathrm{ion}\ \mathrm{state}\end{array}}{\underbrace{k_{p2f}{C}^{diss,p}\left({C}_{sat}^{diss,f}-{C}^{diss,f}\right)}}-\underset{\begin{array}{l}\mathrm{cellular}\  \mathrm{uptake}\  \mathrm{of}\  \mathrm{free}\  \mathrm{ions}\  \mathrm{from}\\ {}\mathrm{the}\  \mathrm{liquid}\  \mathrm{medium}\end{array}}{\underbrace{\frac{D_{12}{SA}_2}{V}\frac{\left({C}^{diss,f}-\frac{C^{diss, cell}V/{V}_{cell}}{PC_{21}}\right)}{Dis_2}}},\end{array}} $$

Similarly, the final rate equation for the protein-bound silver ion concentration (*C*^*diss*, *p*^) in the liquid media is,

13 $$ {\displaystyle \begin{array}{l}\frac{dC^{diss,p}}{dt}=\underset{\begin{array}{l}\mathrm{s}\mathrm{low}\  \mathrm{transfer}\  \mathrm{of}\  \mathrm{silver}\  \mathrm{ions}\  \mathrm{from}\\ {}\mathrm{particles}\  \mathrm{to}\  \mathrm{protein}\  \mathrm{bound}\  \mathrm{state}\end{array}}{\underbrace{\frac{k_p\  Area(t)}{V}\ \left(n\cdot {P}_0-{C}^{diss,p}\right)}}+\underset{\begin{array}{l}\mathrm{initial}\  \mathrm{fast}\  \mathrm{transfer}\  \mathrm{of}\  \mathrm{silver}\  \mathrm{ions}\  \mathrm{from}\ \\ {}\mathrm{particles}\  \mathrm{to}\  \mathrm{protein}\  \mathrm{bound}\  \mathrm{state}\end{array}}{\underbrace{\frac{k_{p2}\  Area(t)}{V}\ \left({n}_2\cdot {P}_0-{C}^{diss,p}\right)}}+\underset{\begin{array}{l}\mathrm{binding}\  \mathrm{of}\  \mathrm{ions}\  \mathrm{from}\  \mathrm{solution}\\ {}\mathrm{to}\  \mathrm{protein}\mathrm{s}\end{array}}{\underbrace{k_{f2p}{C}^{diss,f}\left(n\cdot {P}_0-{C}^{diss,p}\right)}}\\ {}\kern4em -\underset{\begin{array}{l}\mathrm{transfer}\  \mathrm{of}\  \mathrm{silver}\  \mathrm{ions}\  \mathrm{from}\  \mathrm{protein}\hbox{-} \\ {}\mathrm{bound}\  \mathrm{state}\  \mathrm{to}\  \mathrm{free}\ \mathrm{ion}\ \mathrm{state}\end{array}}{\underbrace{k_{p2f}{C}^{diss,p}\left({C}_{sat}^{diss,f}-{C}^{diss,f}\right)}}-\frac{D_{12}{SA}_2}{V}\frac{\left({C}^{diss,p}-\frac{C^{diss, cell}V/{V}_{cell}}{PC_{21}}\right)}{Dis_2}\\ {}\kern1.75em \end{array}} $$

And, the total dissolved ion concentration,*C*^*diss*^, is then calculated as,

14 $$ {C}^{diss}={C}^{diss,p}+{C}^{diss,f} $$

#### Equation for the rate of change in particle size due to dissolution

Based on the empirical dissolution model (Eqs. 9 and 10), the rate at which the mass (*m*_*p*_ = *ρ*_*p*_*V*_*p*_) of a particle changes with time can be written as

15 $$ \frac{d\left({\rho}_p{V}_p\right)}{dt}=-{k}_f{A}_p\left({C}_{sat}^{diss,f}-{C}^{diss,f}\right)-{k}_p{A}_p\left(n\cdot {P}_0-{C}^{diss,p}\right)-{k}_{p2}{A}_p\left({n}_2\cdot {P}_0-{C}^{diss,p}\right). $$

where, *A*_*p*_is defined as the surface area of spherical particles of diameter, *D*_*p*_. If the particle is an aggregate or agglomerate, its surface area is calculated based on the total surface area of the primary particles, i.e.,

16 $$ {A}_p=\pi \times {D}_p^2={N}_{p1}\times \pi \times {D}_{p1}^2,\kern0.5em {D}_p>{D}_{p1} $$

where *D*_*p*1_is the initial diameter of the primary particle, and *N*_*p*1_is the number of primary particles that form a mass equivalent to that of the agglomerate particle.

If *m*_*p*_, *ρ*_*p*_, and *V*_*p*_is the mass, density, and volume of the agglomerate particle, respectively, then for an agglomerate particle,

17 $$ {m}_p={\rho}_p{V}_p={N}_{p1}{\rho}_{p1}{V}_{p1}, $$

where *ρ*_*p*1_ and *V*_*p*1_ are the density and volume of the primary particle, respectively. Given that ${V}_p=\frac{\pi {D}_p^3}{6}$ and ${V}_{p1}=\frac{\pi {D}_{p1}^3}{6}$, and from Eq. 16, we have

18 $$ {N}_{p1}=\frac{\rho_p{V}_p}{\rho_{p1}{V}_{p1}}=\frac{\rho_p{D}_p^3}{\rho_{p1}{D}_{p1}^3}, $$

which can be substituted in Eq. 16 to calculate the surface area of agglomerate particles.

Based on Eqs. 15–18, the final equation for the rate of change in particle size due to dissolution can be written as,

19 $$ \frac{d\left({D}_p\right)}{dt}=-\frac{2}{\rho_{p1}}a\left({k}_f\left({C}_{sat}^{diss,f}-{C}^{diss,f}\right)+{k}_p\left(n\cdot {P}_0-{C}^{diss,p}\right)+{k}_{p2}\left({n}_2\cdot {P}_0-{C}^{diss,p}\right)\right); $$

*a* = *D*_*p*_/*D*_*p*1_ for agglomerates (*D*_*p*_ > *D*_*p*1_) and 1 for particles of size *D*_*p*_ ≤ *D*_*p*1_.

#### The final equation for the particle number density in media

The final equation for the particle density in the liquid media is obtained by substituting $\frac{d\left({D}_p\right)}{dt}$in Eq. 1 with the right-hand side expression of Eq. 19, which is

20 $$ {\displaystyle \begin{array}{l}\frac{\partial N\left({D}_p;x,t\right)}{\partial t}={D}_{diff}\left({D}_p\right)\frac{\partial^2N\left({D}_p;x,t\right)}{\partial {x}^2}-{V}_t\left({D}_p\right)\frac{\partial N\left({D}_p;x,t\right)}{\partial x}\\ {}+\frac{2}{\rho_p}\left({k}_f\left({C}_{sat}^{diss,f}-{C}^{diss,f}\right)+{k}_p\left(n\cdot {P}_0-{C}^{diss,p}\right)+{k}_{p2}\left({n}_2\cdot {P}_0-{C}^{diss,p}\right)\right)\frac{\partial N\left({D}_p;x,t\right)}{\partial {D}_p}\end{array}} $$

The variables, *D*_*p*_and *ρ*_*p*_ in Eq. 20 will refer to the diameter and density of the primary particle for *D*_*p*_ ≤ *D*_*p*1_and of the agglomerate particle for*D*_*p*_ > *D*_*p*1_. If there are particles of sizes greater than the initial size of the primary particle and if they are not agglomerates, but a primary particle coated with a protein layer, of thickness,Δ*R*_*c*_, then *D*_*p*_ and *ρ*_*p*_ in Eq. 20 will refer to the diameter and density of the primary particle. But the diffusivity and sedimentation velocity will be evaluated based on the density,*ρ*_*pc*_, and the diameter, *D*_*pc*_(=*D*_*p*_ + 2Δ*R*_*c*_) of the protein-coated particle; that is, *D*_*diff*_(*D*_*pc*_)and *V*_*t*_(*D*_*pc*_) will replace *D*_*diff*_(*D*_*p*_) and *V*_*t*_(*D*_*p*_) in Eq. 20. However, the mass, concentration, surface area, and volume of the particles (agglomerate or protein-coated) will always be calculated based on the diameter and the density of the primary particle.

#### Boundary equations for the particle number density

While the cell uptake term (fourth term in the right-hand side. of Eq. 12) accounts for the cell boundary condition for the free ions, the boundary conditions for the particle number density function are defined as

21a $$ {D}_{diff}\left({D}_p\right)\frac{\partial N\left({D}_p;x,t\right)}{\partial x}-{V}_t\left({D}_p\right)\ N\left({D}_p;x,t\right)=0\kern1em \mathrm{at}\kern0.5em x=0\ \left(\mathrm{top}\ \mathrm{surface}\right), $$

and,

21b $$ N\left({D}_p;x,t\right)=0\kern1em \mathrm{at}\kern0.5em x=L\kern0.5em \left(\mathrm{bottom}\  \mathrm{surface}\right) $$

In this work, the ISD3 simulations were performed assuming that all the particles reaching the cell surface are instantaneously taken up the cells, which is served by the boundary condition in Eq. 21b. Given this boundary condition, a perfect match between ISD3 predictions and experiments should not be expected because it is possible that not all particles may be taken up by the cells. But the purpose of the current boundary condition is to determine the maximum number of particles that could be associated with the cells. By comparing the predicted value for the cell-associated mass and the experimental value for the deposited mass in cells, we will be able to infer whether all or some of the particles were taken up the cells in the experiments. If uptake is not complete then it could alter the rate of deposition due to upward diffusion [15].

#### Initial particle number density function and ion concentration values for the ISD3 simulations

The initial particle number density function, *N*(*D*_*p*_; *x*, 0), depends on the initial size distribution and spatial location of the particles in the liquid medium. The initial size distribution profile can be approximated using a mathematical function (e.g., Gaussian distribution) based on size frequency distribution data. Here, we approximate the distribution based on a formula, such as the Gaussian function. Specifically, if *g*(*D*_*p*_; *x*, 0) is the distribution of the initial number fraction of particles with size less than diameter *D*_*p*_at every axial location *x* (the cumulative number fraction), then

22 $$ N\left({D}_p;x,0\right)=N(0)\frac{dg\left({D}_p;x,0\right)}{dD_p}, $$

where *N*(0) is the initial, total number of particles.

Since *N*(0) =  ∬ *N*(*D*_*p*_; *x*, 0)*dD*_*p*_*dx*,

23 $$ \iint \frac{dg\left({D}_p;x,0\right)}{dD_p}{dD}_p dx=1. $$

Note, $\frac{dg\left({D}_p;x,0\right)}{dD_p}$ is the number fraction density in *x*-*D*_p_ parameter space. If the distribution is a Gaussian distribution with average ($\overline{D_p}$) and standard deviation (*σ*) values for the particle size, then the number fraction density can be approximated as a Gaussian function, *f*(*D*_*p*_):

24 $$ f\left({D}_p\right)=\frac{1}{\sqrt{2\pi}\sigma }{e}^{-\left(\frac{{\left({D}_p-\overline{D_p}\right)}^2}{2{\sigma}^2}\right)}. $$

The initial size distribution of the particles along the liquid column can be assumed to be uniformly or non-uniformly distributed along the height of the liquid column. If the initial size distribution follows a Gaussian distribution in *D*_*p*_ space and uniform distribution in *x*, then the initial number fraction density in *x*-*D*_*p*_ parameter space, denoted as *n*(*D*_*p*_; *x*, 0), can be approximated as

25 $$ n\left({D}_p;x,0\right)=\frac{dg\left({D}_p;x,0\right)}{dD_p}=\frac{f\left({D}_p\right)}{L}. $$

Based on the initial number fraction density,*n*(*D*_*p*_; *x*, 0), and the initial particle mass concentration, *C*(0), the initial particle number,*N*(0), can then be computed as

26 $$ N(0)=\frac{C(0)\times V}{\iint n\left({D}_p;x,0\right){v}_p{\rho}_p{dD}_p dx}, $$

where, *v*_*p*_ and *ρ*_*p*_are the volume and density of particles of diameter *D*_*p*_.

Thus, the initial number density function in *x*-*D*_*p*_ parameter space is computed as

27 $$ N\left({D}_p;x,0\right)=N(0)\times n\left({D}_p;x,0\right). $$

In all the simulations, the initial ion concentration was set to zero in the liquid media and in the cells:

28 $$ {C}^{diss,f}(0)={C}^{diss,p}(0)={C}^{diss, cell}(0)=0. $$

#### Numerical approach for solving the equations of the ISD3 model

The ISD3 model, which is based on the population balance equation (Eq. 20), looks similar to the ISDD model [14], except for the third term (on its right-hand side) that accounts for the change in particle size due to dissolution. Instead of solving for the particle mass concentration (as in ISDD), the ISD3 model solves for particle number density,*N*(*D*_*p*_; *x*, *t*), to simultaneously track changes in both the size and spatial distribution, and the number, of the particles in the liquid medium due to diffusion, sedimentation, and dissolution.

The final rate equations for ions (Eqs. 12 and 13) and particle number density (Eq. 20) are numerically solved along with the boundary (Eqs. 21a and 21b) and initial (Eqs. 24, 25, 27, and 28) conditions to obtain solutions to the particle number density and the ion concentrations. The numerical algorithm used for solving the equations was based on the finite difference method and was implemented in MATLAB – the details are given in the Supporting Information. The numerical solution for the particle number density was then used to calculate the mass, number, size distribution and surface area of particles, both in the liquid media and in the cells; the formulas for these calculations are given below.

#### Particle surface area

The surface area term, *Area*(*t*), which appears in the final equation for the free ion concentration, is defined as the total surface area of the (spherical) particles available for dissolution in the liquid column, at time *t*, and is computed as,

29 $$ Area(t)=\iint N\left({D}_p;x,t\right){A}_p\left({D}_p\right)\ {dD}_p\  dx=\iint N\left({D}_p;x,t\right)\pi {D}_p^2\ {dD}_p\  dx $$

#### Particle number fraction density

The number fraction density at any time, *t*, in *x*-*D*_*p*_ parameter space,*n*(*D*_*p*_; *x*, *t*), is computed by dividing the number density, *N*(*D*_*p*_; *x*, *t*), by the initial number of particles in the liquid medium, i.e.,

30 $$ n\left({D}_p;x,t\right)=\frac{N\left({D}_p;x,t\right)}{N(0)} $$

#### Mass and number concentration of particles in the liquid medium

The mass concentration of particles in the liquid medium is defined as the mass of particles in unit volume of physical space. It is calculated from the number density distribution, *N*(*D*_*p*_; *x*, *t*), as

31 $$ C\left(x,t\right)=\frac{1}{A}\underset{D_p}{\int }N\left({D}_p;x,t\right){v}_p{\rho}_p{dD}_p, $$

where, *A* is defined as the surface area of the cells, and is considered equivalent to the surface area of the bottom surface of the liquid column.

The number concentration of particles in the liquid medium is defined as the number of particles in unit volume of physical space. It is calculated from the number density distribution, *N*(*D*_*p*_; *x*, *t*), as

32 $$ {C}_N\left(x,t\right)=\frac{1}{A}\underset{D_p}{\int }N\left({D}_p;x,t\right){dD}_p. $$

#### Number size distribution of particles deposited in cells

The number distribution of particles deposited in the cells, *N*^*cell*^(*D*_*p*_; *t*), is obtained by integrating the following equation,

33 $$ \frac{dN^{cell}\left({D}_p;t\right)}{dt}=-\frac{dN\left({D}_p;t\right)}{dt}+\int \left[\frac{\partial }{\partial {D}_p}\left(N\left({D}_p;x,t\right)\frac{dD_p}{dt}\right)\right] dx $$

From the *N*^*cell*^(*D*_*p*_; *t*), the mass and surface area of particles in the cell media are calculated.

### Model parameters

Model parameters are measured or optimized as described in the methods and results sections. A full list of model parameters is found in Table 1.

**Table 1:** ISD3 simulation parameters for the 20 nm and 110 nm systems

Parameters	20 nm system	110 nm system	Fitted Parameter?
Liquid media characteristics			
Media height, *L* (m)	0.00315	0.00315	No
Media volume, *V* (mL)	3	3	No
Media temperature, *T* (K)	310	310	No
Media viscosity, *μ*(N s/m^2^)	0.00074	0.00074	No
Media density, *ρ*_*f*_ (g/mL)	1	1	No
Surface area, *A* (m^2^)	0.000952	0.000952	No
Initial particle characteristics			
Particle state	Primary particles coated with proteins	Primary particles coated with proteins	
Primary particle size / diameter, *d*_*p*_ (nm)	20	110	No
Primary particle density, *ρ*_*p*_(g/cm^3^)	10	10	No
Thickness of protein layer, Δ*R*_*c*_(nm)	12	22.5	No
Effective diameter, *d*_*pc*_ = *d*_*p*_ + 2Δ*R*_*c*_(nm)	44	155	No
Effective density, *ρ*_*pc*_(g/cm^3^)	1.583	1.914	No
Numerical grid spacing and time discretization			
Grid spacing along particle diameter, Δ*D*_*p*_(nm)	1	1	No
Grid spacing along media height, Δ*x* (m)	3.1532E-6	3.1532E-6	No
Total simulation time, *t*_max_ (h)	24	24	No
Parameters of the dissolution model			
*P*_0_(FBS %)	10	10	No
Rate constant for the transfer of ions from the particle surface to the free ion state, *k*_*f*_(mL nm^− 2^ h^− 1^)	6.00E-18	6.00E-18	Yes
Saturated concentration of free ions in solution, ${C}_{sat}^{diss,f}$(μg/mL)	1	1	Yes
Rate constant for the slow transfer of ions from the particle surface to the proteins, *k*_*p*_(mL nm^− 2^ h^− 1^)	3.00E-17	3.00E-17	Yes
Rate constant for the initial fast transfer of silver ions from the particle surface to the protein-bound state, *k*_*p*2_(mL nm^− 2^ h^− 1^)	1.00E-15	1.00E-15	Yes
Rate constant for the transfer of free ions from solution to the protein-bound state, *k*_*f*2*p*_(ug^− 1^ mL h^− 1^)	0.0114	0.0114	Yes
Rate constant for the transfer of ions from the protein-bound state to the free ion state, *k*_*p*2*f*_(ug^− 1^ mL h^− 1^)	0.016	0.016	Yes
*n* (μg mL^−1^ FBS%^− 1^)	0.4	0.4	Yes
*n*_2_(μg mL^− 1^ FBS%^− 1^)	0.19822	0.19822	Yes
Parameters of the cell uptake model for ions			
Diffusion coefficient, *D*_12_(cm^2^/h)	9.02e-9	9.02e-9	Yes
Cell membrane thickness,*Dis*_12_(cm)	7.8e-7	7.8e-7	Yes
Cell:media silver ion partition coefficient, *PC*_21_	25.8	25.8	Yes
Cell volume, *V*_*cell*_(mL)	1.936e-3	1.936e-3	No

### Modeling varying size and spatial distribution of particles

The time-dependent solutions for the size and spatial distribution of particles in the liquid media will depend on the initial size distribution. Since experimental data suggested that the initial size distribution of the 20 and 110 nm primary silver nanoparticles were nearly monodisperse, it was numerically described as a Gaussian function with all particles having a mean diameter equal to the effective diameter, i.e., of the protein-coated particles. But the application of the ISD3 model is not limited to using a specific mathematical function for the initial size distribution. The distribution can be described using any function representative of the experimental data (e.g., size frequency distribution data) or it can be the data itself. The ISD3 model not only solves for the size but also for the spatial distribution of the particles along the height of the liquid column. The population balance formalism of the ISD3 model enables the representation of both distributions as a particle number density, (*N*(*D*_*p*_;*x*,*t*)), which is a function of the location (*x*) and particle diameter (*D*_*p*_), with the protein thickness excluded. The initial particle number density as a function of spatial location is not known; therefore, it is typically assumed to be uniformly distributed along the height of the liquid column (as done for the current simulations), since the particles are expected to be completely mixed in solution. Theoretically, one can assume any spatial distribution of the particles. For example, the particles may be considered located on the top surface or at the center of the liquid column; or, the number of particles may be normally distributed about the center of the liquid column for each particle size range. Thus, generally, any initial size and spatial distribution of the particles can be used, as long as they are relevant to the in vitro particulate system.

If the mean and standard deviation values are based on a normal or log-normal distribution of particle sizes, then the respective formula of the distribution function can be used to construct the initial size distribution for the ISD3 simulations. If the form of the distribution is neither normal nor log-normal, then experimentally derived data has to be directly used to construct the initial solution. But the initial size range of the distribution cannot theoretically extend below the primary particle size because no dissolution has occurred yet and the possibility of particles breaking up is neither expected nor considered. Therefore, if a normal or a log-normal distribution is used, then that part of the distribution below the primary particle diameter has to be discarded. The upper-limit of size distribution data can be set to any value (e.g. it could be 3 standard deviations above the average value). Whatever value is used, it should correspond to that of the particles. For instance, in complex mixtures of particles in FBS media, the whole size distribution profile need not be of the particles, since there are other components in the media (e.g., proteins) of sizes smaller and larger than the particles. Therefore, the appropriate cut-off for the upper size limit has to be used while the primary size can serve as the lower cut-off value. And, the resulting distribution has to be normalized before it is used in the ISD3 simulations. It is noted that initial solution is an approximation of the actual distribution, and does not have to exactly match experimental data, as long as the accuracy of the predictions is not significantly affected.

It is noted that the simulations were done with the criteria that particles do not dissolve below 10 nm; this criteria was used to limit dissolution only to silver and to keep the gold core intact (the gold core is about 7 nm in diameter). In this way, the number of particles is also conserved in all the simulations.

## Results

ISD3 is a specific implementation of the more general Population Balance Equation (PBE)^3^ with elements for dissolution and cell uptake. The size and density of silver nanoparticles in the experimental media were measured directly. The cell uptake and dissolution models were developed and integrated into the population balance equation to create ISD3. The two kinetic models for particle dissolution and uptake of ions by cells were calibrated against experimental particle dissolution and uptake data. Specifically, the dissolution submodel was calibrated to experimental data on the rate and extent of dissolution of 20 nm silver nanoparticles (12.5 μg/ml) in media containing 1, 10 and 30% FBS. The resulting dissolution model was then tested against dissolution time-course data for 20 and 110 nm silver nanoparticles at multiple concentrations in media containing 10% FBS. The cell ion uptake submodel was calibrated against ion-only cell uptake time course data. Finally, the full ISD3 model was tested against cell silver concentration time-course data following exposure to 20 and110 nm silver nanoparticles. Results are presented in that order.

### Size and density of silver Nanoparticles in RPMI + FBS

#### Silver particle size and density

The sizes of the nominally 20 and 110 nm nanoparticles, measured by DLS in 10% FBS + RPMI solution, were 44 and 155 nm, respectively. Without corresponding effective density measurements, these data may be interpreted to indicate some level of agglomeration, especially for the 20 nm particles. Our density measurements using the VCM in 10% FBS + RPMI indicate that the particles were 80.86 (20 nm) to 84.17% (100 nm) lighter than silver, (10.49 g/cm^3^) 1.583 and 1.914 g/cm^3^, respectively. Such low density values are unrealistic of agglomerates too small to entrap water, but may result from the addition of low density proteins to the particle surface. This existence of a protein corona on the surface of the 20 and 110 nm particles has been reported in the literature [27, 40]. Therefore, in all our simulations, the 20 nm and 110 nm particles were modeled as primary particles coated with a protein layer of thickness, 12.0 and 22.5 nm, respectively. The thickness of the protein layers was set to ½ the difference in diameters measured by TEM and DLS, producing an effective particle size equal to the value measured by DLS. This value and the measured density was fixed for the duration of all simulations.

In the case of sedimentation and diffusion, the size and density of the particles correspond to the effective diameter and density of the protein-coated particles, which is 44 nm and 1.583 g/ cm^3^ for the 20 nm system, and 155 nm and 1.914 g/ cm^3^ for the 110 nm system.

For purposes of calculating masses, volumes and numbers of particles, the diameter measured by TEM and the density of silver are used throughout this manuscript. For purposes of modeling diffusion and sedimentation, the density of the protein coated particle and the diameter of the protein covered particle measured by DLS are used.

### Calibration of the dissolution and cell uptake kinetic models

#### Experimental evidence of particle and protein concentration effects on dissolution

**Fig. 2:**
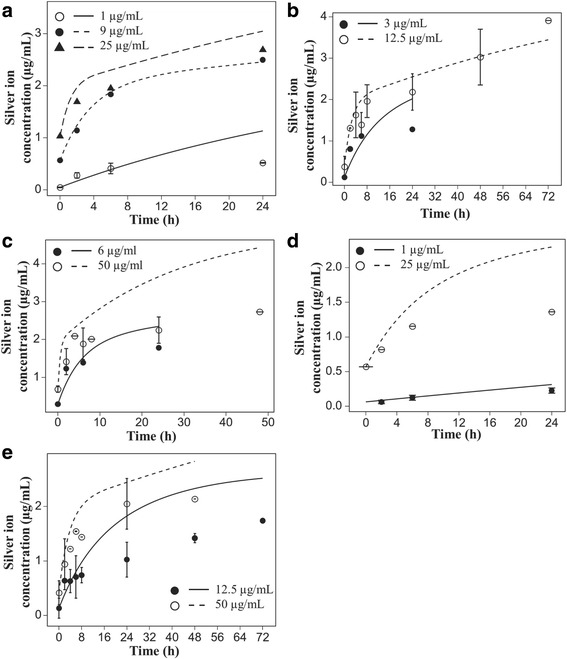
Dissolution of silver nanoparticles in 10% FBS. Dissolution time-course for 20 (**a** – **c**) and 110 (**d** and **e**) nm silver particles in 10% FBS. Lines represent model predictions based on parameters fitted using the 20 nm silver dissolution data (Fig. 3). Points represent experimental data

To identify the proper form of the dissolution model, the rates and extent of silver nanoparticle dissolution were determined for 20 and 110 nm particles in media containing varying levels of FBS and varying particle concentrations (1, 3, 6, 9, 12.5, 25, and 50 μg/mL). Media ion concentrations increased over time for all particles and concentrations (Data points in Fig. 2a-e). Particle dissolution was a biphasic process, consisting of an initial fast rate followed by a slower rate as ion concentrations approached apparent saturation points. Media ion concentrations did not reach full saturation within the 24–72 h experimental period. Final ion concentrations were dependent on the initial particle concentration.

**Fig. 3:**
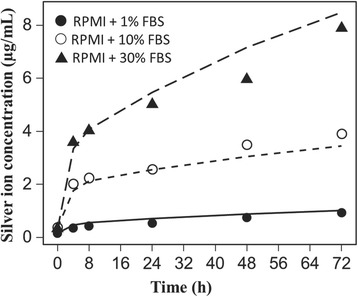
Effect of serum proteins levels on silver nanoparticle dissolution kinetics. Dependence of initial rates of 20 nm silver nanoparticle dissolution and total dissolved silver ion concentrations on serum protein levels. Lines represent model fits to experimental data (points)

We hypothesized that that the biphasic dissolution curves reflected some influence of serum proteins, which contain sulfhydryl groups that reversibly bind with silver ions. Increasing the media content from 1, to 10 and then 30% FBS increased both the initial fast rate of dissolution of 20 nm particles (12.5 μg/ml) and the total dissolved ions in the media (Fig. 3). We interpreted these data as evidence of serum proteins serving as a significant sink in cell culture media affecting the kinetics of dissolution. While the serum proteins were found to affect the dissolution rate of silver nanoparticles in the systems we studied, different proteins can have different effects on the release of silver ions [41].

The experimental dissolution data were consistent with a description of dissolution that included not only a dependence on particle concentration and transfer of ions from the particle surface to the free ion state in the media, but also the transfer ions to the protein-bound state.

#### Calibration of the dissolution model

The eight ISD3 model parameters related to silver particle dissolution (Eqs. 12 and 13) were fitted to total media silver ion concentration time-course data for 20 nm silver particles in RPMI media with 1%, 10%, and 30% FBS (Fig. 3).

We elected to obtain a single set of dissolution model parameters to fit the total silver ion concentrations for 20 and 110 nm particles, across all concentrations and time. The dissolution parameters calibrated to the 20 nm particle dissolution data were retained and applied to all other particle size and concentration time course data with acceptable error, and good agreement with two phases of dissolution observed: an early rapid phase followed by a slower phase approaching equilibrium (Simulation lines, Fig. 2).

Table 1 lists the parameter values that were obtained by fitting Eqs. 9 and 10 to the dissolution data of the 20 nm silver particles in 1%, 10%, and 30% FBS. The fitted *n*_*2*_ parameter was found to vary with *n* and *P*_0_ as${n}_{2c}\times n\times {P}_0^{-0.403}$, where *n*_2*c*_ = 1.2534. Table 1 shows the *n*_*2*_ value for *P*_0_ = 10. The pre-factor, *n*_2*c*_, was selected as the parameter instead of *n*_*2*_ in the parameter identifiability and sensitivity analysis.

To determine which of the 8 parameters were identifiable based on the fitted data, we performed a collinearity analysis using the *colin* function from R’s FME package. The collinearity indices are plotted as a function of the number of selected parameters in Additional file 1: Figure S1. Parameter sets with collinearity indices less than 20 are typically considered as identifiable. As seen in Additional file 1: Figure S2, the maximum number of identifiable parameters is 6. The six identifiable parameters corresponding to the lowest collinearity index value (11.7) are ${C}_{sat}^{diss,f}$, *k*_*f*_, *k*_*f2p*_, *k*_*p*2f_, *k*_*p*_, *k*_*p2*_, and *n*_2*c*_.

We also performed a sensitivity analysis (using *sensRange* function from R’s FME package) by varying each individual parameter within 10% of their fitted value. The results are shown in Additional file 1: Figure S3. The total silver ion concentration was not sensitive to 10% changes in the *k*_*f*_ and *k*_*f2p*_ at all FBS concentrations. The model was also not sensitive to *k*_*p*2f_, although slight sensitivity was observed for the total dissolved silver in 1% FBS. The sensitivity range of total dissolved silver to the other parameters can be seen by the shaded regions in Additional file 1: Figure S3. Additional file 1: Figure S4 shows the sensitivity of the model when all the parameters were allowed to vary within 10% of their fitted values. The results indicate that the model is robust enough in describing the dissolution behavior of silver nanoparticles in RPMI + FBS media, even within the 10% sensitivity range of the parameters.

The initial condition for the fraction of media ions bound to protein was set to 0.85, generally reflecting the high ratio of protein-bound to free ions observed in the media containing only silver ions. Decreasing the fraction of protein-bound ions improves the model predictions for the total dissolved silver in 1% FBS media, but not so much in the 10 and 30% FBS media (Additional file 1: Figure S1).

As shown in Fig. 2a-e (Simulation lines), the single set of parameters provided simulations of all the silver nanoparticle dissolution data consistent with a) two rates of dissolution, and b) particle surface area dependent dissolution. Simulations generally agreed well with the concentration time course data. In some cases, model simulations differed as much as 100% from measured concentrations, typically at the later time points.

#### Calibration of the cell ion-uptake model

The parameters of the cell ion-uptake model were fitted to silver ion uptake time-course data in RAW 264.7 macrophage cells exposed to 0.5 and 1.5 μg/mL silver ions in RPMI + 10% FBS.

**Fig. 4:**
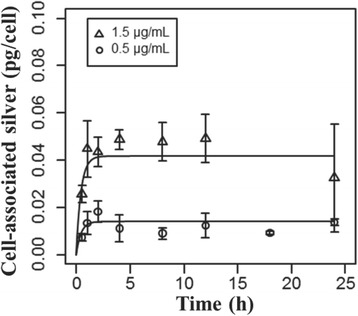
Cell uptake kinetics of silver ions. Levels of silver associated with RAW 264.7 cells exposed to 0.5 (circles) or 1.5 μg/mL (triangles) silver ions (silver acetate) over time. Lines are model fits to the data (points)

The observed uptake of silver ions from cell culture media was rapid, reaching a steady state level proportional to the media silver ion concentration (Fig. 4). The initial uptake rate was consistent with diffusion limited entry, not instantaneous partitioning. The uptake of silver ions was described in ISD3 as diffusion-limited uptake, bounded by the measured cell:media silver ion partition coefficient. The measured cell volume, and thickness of the cellular membrane (for diffusion calculations) can be found in Table 1. The partition coefficient and diffusion coefficient were fit to the time course of silver levels in RAW 264.7 cells after exposure to silver ions at 0.5 and 1.5 μg/mL (Fig. 4). A single set of fitted parameters (Table 1) accurately described the time-course of cellular uptake of silver ions in cell culture media. The cell:media silver ion partition coefficient was 25.8.

### ISD3 comparison to measured Total silver in cells exposed to silver Nanoparticles

The final set of parameters used in the ISD3 simulations are listed in Table 1.

**Fig. 5:**
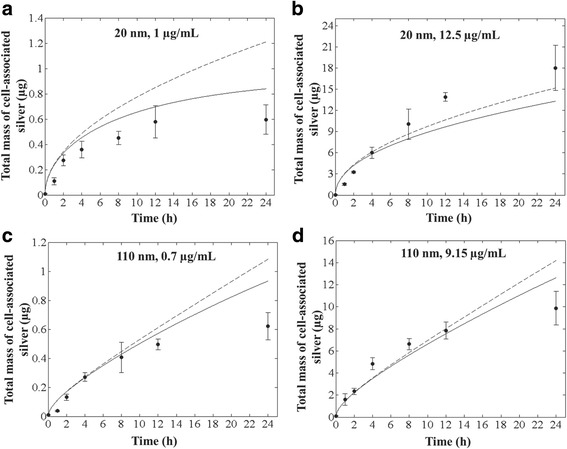
ISD3 predictions for total deposited silver. Comparison between ISD3 (black line) and experimental (black circles with error bars) values for total mass of silver in cells versus time for systems with initial concentration and diameter: (**a**) 1 μg/mL and 20 nm; (**b**) 12.5 μg/mL and 20 nm; (**c**) 0.7 μg/mL and 110 nm; and, (**d**) 9.15 μg/mL and 110 nm. The dashed curve corresponds to the simulation result without dissolution

To test the ability of ISD3 to describe cellular dosimetry of combined particle and ion uptake in a mixed particle-ion system based solely on first principles of diffusion and sedimentation and calibrated submodels for cellular uptake of ions and particle dissolution, simulations were compared to experimentally measured total silver concentrations. ISD3 accurately described the total cellular content of silver following exposure to 1 and 12.5 μg/mL, of the 20 nm particles (Fig. 5a and b); and, for two initial concentrations, 0.7 and 9.15 μg/mL, of the 110 nm particles (Fig. 5c and d). The discrepancy between measured and predicted total cell silver content was greatest at 24 h, but overall it was within a deposited dose fraction of 0.15.

The preponderance of cell-associated silver is predicted to be initially of particle origin, not ions taken up by cells. Ions appear to form only about 0.46–3.26% of the total silver mass in cells. For example, at the end of 24 h, the predicted total mass of ions in cells from exposure to 1 μg/mL (3 μg) or 12.5 μg/mL (37.5 μg) of 20 nm particles was 0.02826 and 0.11323 μg, respectively. Similarly, the predicted total mass of silver ions present in cells after exposure to 0.7 μg/mL (2.1 μg) or 9.15 μg/mL (27.45 μg) of 110 nm particles was 0.00599 and 0.05788 μg, respectively.

Dissolution of particles was predicted to affect the delivery of the dominant form of silver, nanoparticles, to cells (Fig. 5). Without dissolution (dashed lines, Fig. 5), ISD3 over predicted the total silver content of cells after 24 h. At earlier time points, where dissolution had a smaller impact on particle size, there was only minor differences in projected particle delivery. We interpret these findings as evidence that in some cases, it is necessary to account for dissolution of particle during experiments to properly simulate total cellular dose of soluble metal nanoparticles.

### ISD3 comparison to measured media silver ions and Nanoparticles

**Fig. 6:**
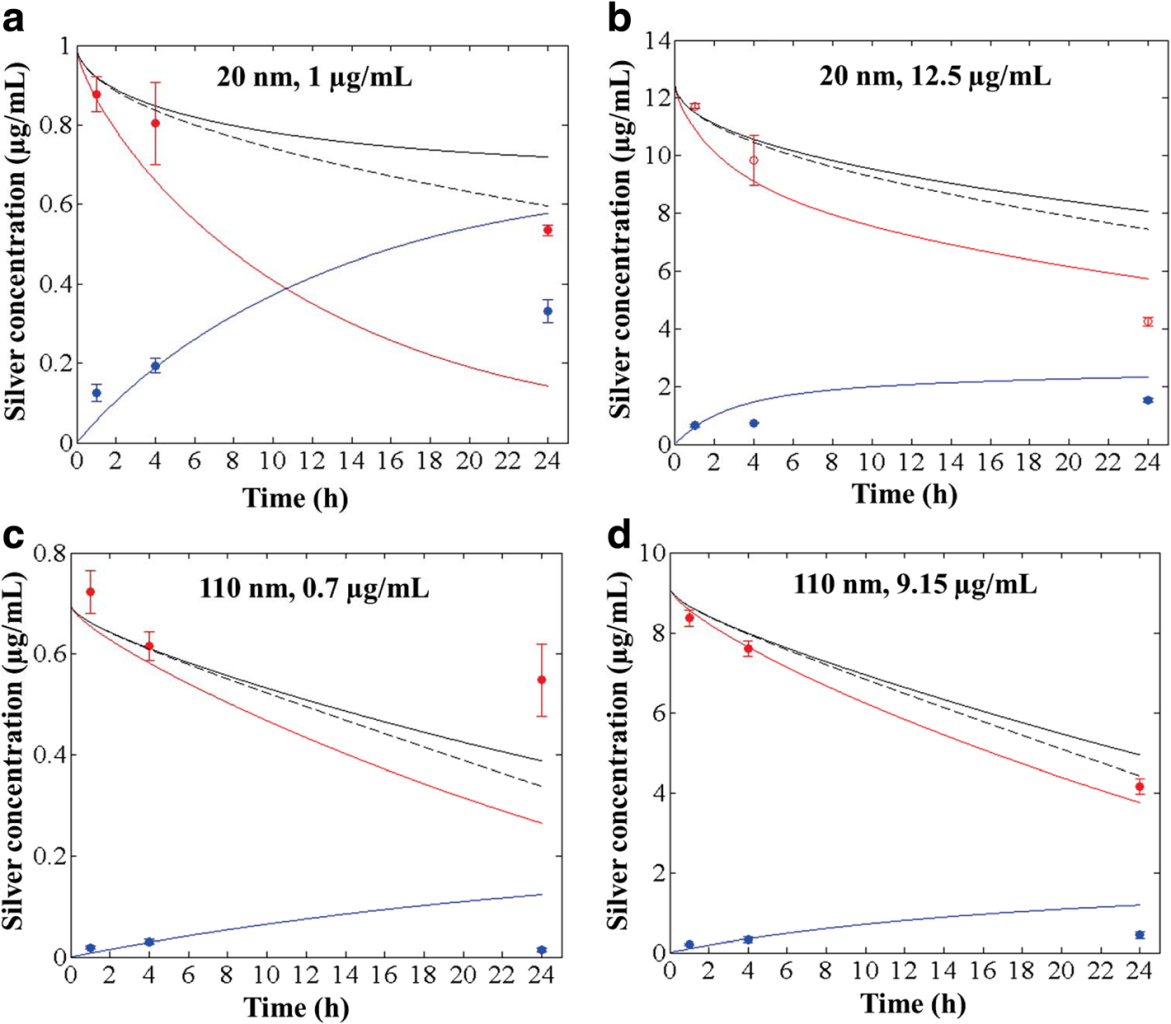
ISD3 predictions for the concentration of silver nanoparticles and ions in the liquid media. Comparison between ISD3 results (solid lines) and experiments (symbols) for total silver concentration (black), silver nanoparticle concentration (red) and silver ion concentration (blue) in the liquid column as a function of time. Results are shown for four systems with initial particle concentration and diameter: 1 μg/mL and 20 nm (**a**); 12.5 μg/mL and 20 nm (**b**); 0.7 μg/mL and 110 nm (**c**); and, 9.15 μg/mL and 110 nm (**d**). The black dashed line corresponds to the total particle concentration of silver, predicted in the absence of dissolution

**Fig. 7:**
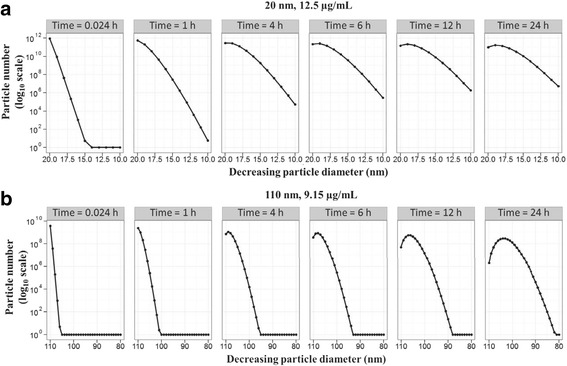
ISD3 predictions for the number and size of cell-associated silver nanoparticles. Snapshots of cell-associated number of particles as a function of diameter (*D*_*p*_) for (**a**) 20 nm and (**b**) 110 nm particles. The initial concentration of 20 and 110 nm particles in the liquid media is 12.5 and 9.15 μg/mL, respectively

While cellular dosimetry was the main focus of this work, we also evaluated the ability to accurately describe the concentrations of ions and particles in media over time as additional confirmation of the general accuracy of ISD3 description of dissolution and delivery,

Given the measurement errors, there was fairly good agreement between the predicted and measured concentrations of silver particles and total silver in the media, at the two early time points (1 and 4 h), both at the low and at the high initial concentrations of the 20 and 110 nm particles. The agreement in the particle concentration was within an absolute percentage difference of 1 and 18% (1 μg/mL, 20 nm – Fig. 6a; 7 and 8% (12.5 μg/mL, 20 nm – Fig. 6b); 9 and 5% (0.7 μg/mL, 110 nm – Fig. 6c; and, 2 and 0% (9.15 μg/mL, 110 nm – Fig. 6d), at the 1 and 4 h time points, respectively. The corresponding differences in total silver were 8 and 5% (1 μg/mL, 20 nm); 7 and 0% (12.5 μg/mL, 20 nm); 11 and 6% (0.7 μg/mL, 110 nm); and, 1 and 1% (9.15 μg/mL, 110 nm), respectively. There was also excellent agreement between the predicted and measured concentrations at 24 h for the higher exposure experiment with 110 nm particles (Fig. 6d): 9 and 7% difference in particle concentration and total silver, respectively. There was also fair agreement between the predicted and measured silver ion concentrations at the early time points. There was significant divergence between predicted and observed silver ion and silver particle concentrations after 24 h exposures to both particle sizes at the lower concentrations, 1 and 0.7 μg/mL.

Simulations of total silver in media deviated further from experimentally measured values when dissolution of particles was not considered (Fig. 6). We interpret this as further evidence that accounting for dissolution of silver particles, and other soluble nanoparticles may be important for understanding dosimetry in liquid test systems.

#### Dissolution effects on particle number and size distributions

##### Particle delivery to cells

Particle dissolution leads to a time-dependent change in particle size in the media and in the size class distribution of particles presented to and taken up by cells. The total amount of mass deposited will depend on the size distribution of the particles in the cells. Figure 7a and b show how the number of particles of various sizes (*D*_*p*_) delivered to cells changes for systems that initially contained 12.5 μg/mL of 20 nm particles and 9.15 μg/mL of 110 nm particles in the liquid media, respectively. Particle sizes in the cells range from 20 to 10 nm for the system that started with 20 nm particles (Fig. 7a), and from 110 to 84 nm for the system that started with 110 nm particles (Fig. 7b). The size distribution of the particles in the cells is related to the size distribution of the particles in the liquid media, because, the rates at which particles dissolve, diffuse, and settle, will vary across the various particle size classes in the liquid media.

##### Media particle size distributions

**Fig. 8:**
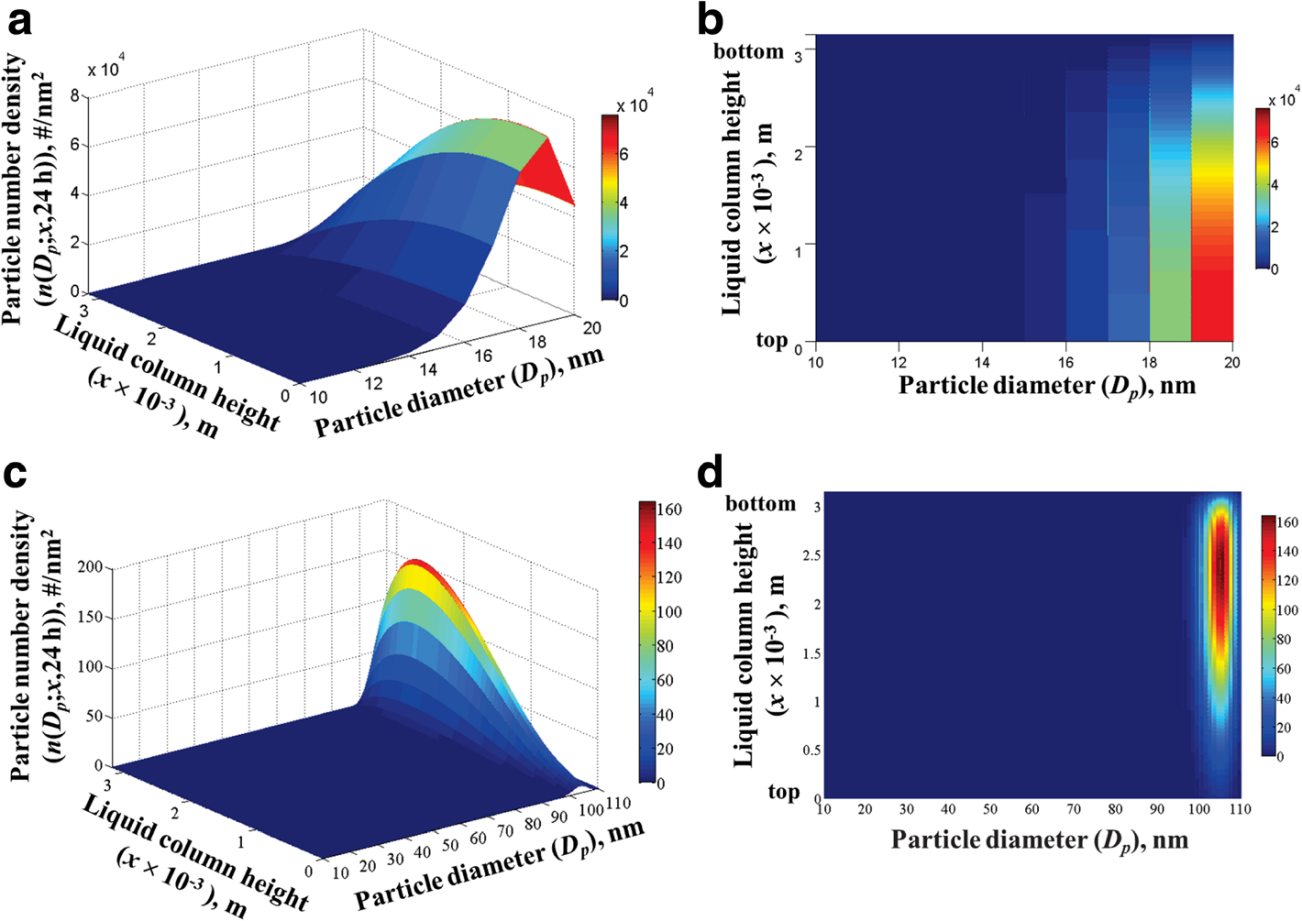
ISD3 predictions for the number density of silver nanoparticles in the liquid media. 3D and 2D mesh plots of particle number density as a function of liquid column height (*x*) and particle diameter (*D*_*p*_) after 24 h, starting with 12.5 μg/ml of 20 nm silver particles (**a** and **b**), and with 9.15 μg/ml of 110 nm silver particles (**c** and **d**)

Because the transport rates will vary for particles of different sizes, the number of particles in the liquid media is expected to be non-uniformly distributed in size and along the liquid column. This effect of dissolution on the size and spatial distribution of particles in the liquid media can be clearly understood from the particle number density distribution in the *x*-*D*_*p*_ parameter space, where *x* is the height of the liquid column, and *D*_*p*_ is the particle diameter (excluding the protein layer). As an example, Fig. 8 represents the number density profiles at 24 h, of systems that initially had 12.5 μg/mL concentration of 20 nm particles (Fig. 8a and b) and 9.15 μg/mL concentration of 110 nm particles (Fig. 8c and d). As seen in the figures, the size distribution at 24 h is non-uniform in *x* and *D*_*p*_, and is completely different from the initial size distribution. The zero values at *x* = 0.00315 m reflects the boundary condition at the bottom of the liquid column, where all particles are considered to disappear from the system into the cells. As seen in the figures, there is a flux of particles towards decreasing sizes (due to dissolution) and down the liquid column (due to sedimentation and diffusion). Clearly, the distribution profiles are different between the 20 nm and 110 nm particle systems, because of differences in the initial size distribution, concentration, and density of the particles in both systems.

The initial size distribution contained particles of one size (20 or 110 nm plus the respective protein layer thickness) at all axial locations. However, for the 20 nm system, the 24-h size distribution shows particles of sizes, ranging from 10 to 20 nm. This is elucidated in Additional file 1: Figure S5(a), which shows the actual number of particles (obtained by integrating the number density, *N*(*D*_*p*_; *x*, *t*) along *x*) across all the size diameters, and at selected time points. Initially, there are about 8.9525×10^11^ particles of size *D*_*p*_ = 20 nm, corresponding to 12.5 μg/mL concentration. After 24 h, this number reduces to 9.7053×10^10^ due to dissolution in the liquid media, and due to deposition in cells. For the 110 nm system, the 24-h size distribution shows particles of sizes ranging from 78 to 110 nm – Additional file 1: Figure S5(b). The number of 110 nm particles decreased from 3.9388×10^9^ at time, *t* = 0 to 2.0788×10^6^ at *t* = 24 h. Because the particles in the 110 nm particle system are larger and denser than the particles in the 20 nm system, they will dissolve slower but move down the liquid column faster than the particles of the 20 nm system.

##### Particle sedimentation and diffusion rates

The movement of particles down the liquid column is driven by sedimentation and diffusion. Comparing the number density profiles of the 20 nm and 110 nm systems in *x*-*D*_*p*_ space (Fig. 8), we can notice that the peak in the number density of the 20 nm system tends to spread out downward; whereas, the peak of the 110 nm system tends to move downward faster than it spreads out. This difference reflects differences in the sedimentation rates and diffusion rates of the particles between the two systems. To know which of the two processes, sedimentation or diffusion, dominates the downward movement of the particles, we show in Additional file 1: Figure S6, a plot of the dimensionless Péclet number, *Pe*, versus particle diameter (excluding the protein thickness), *D*_*p*_, for the two systems. The Péclet number for particle movement down the height of the liquid column, *L*, is defined as the ratio of the diffusion time scale to the sedimentation time scale, i.e., $Pe\left({D}_{pc}\right)=\frac{L^2/{D}_{diff}\left({D}_{pc}\right)}{L/{V}_t\left({D}_{pc},{\rho}_{pc}\right)}=\frac{L\times {V}_t\left({D}_{pc},{\rho}_{pc}\right)}{D_{diff}\left({D}_{pc}\right)}$ . The Péclet number,*Pe*(*D*_*pc*_), characterizes how fast a particle of diameter, *D*_*pc*_ (=*D*_*p*_ + 2 × Δ*R*_*c*_) and density, *ρ*_*pc*_, will move downwards by sedimentation compared to diffusion, or vice-versa. Note the density of the protein-coated particles is 1.583 g/cm^3^ and 1.914 g/cm^3^, in the 20 and 110 nm systems, respectively. And, the actual size of the particles, *D*_*pc*_, is equal to *D*_*p*_ + 12 nm and *D*_*p*_ + 22.5 nm in the 20 nm and 110 nm systems, respectively. When *Pe* < < 1, diffusion is dominant and the particles will spread out (diffuse) faster than they will settle (sediment) downwards in time. When *Pe* > > 1, sedimentation is dominant and the particles will spread out slower than they will settle downwards in time. From Additional file 1: Figure S5, we learned that the particle sizes in the 20 nm and in the 110 nm systems, range from 10 to 20 nm and from 84 to 110 nm, respectively. For the respective size ranges, we can find from Additional file 1: Figure S6 that, diffusion dominates the movement of the particles in the 20 nm system (*Pe* < 1) during the whole 24 h, while sedimentation dominates the particle movement in the 110 nm system (*Pe* > 1). Hence, the reason for the peak in the number density profile of the 20 nm system to spread out faster than it moves downward is due to diffusion. And the peak in the number density profile of the 110 nm system tends to move downward faster than it spreads out due to sedimentation.

## Discussion

ISDD is now a widely used platform for describing and predicting the particokinetics and cellular dosimetry of nonsoluble particles in liquid systems [14, 18, 22, 37, 42–49]. Combined with experimental measures of the effective density of agglomerates [18, 22], the accuracy of ISDD has been verified for a wide range of monomeric and polydisperse particles, across particle size and density, including agglomerates [18, 22]. Particokinetic models applying the same general principles, but for example different assumptions regarding nature of particle and liquid packing within agglomerates have also emerged, but have been validated for a smaller subset of high-density, rapid settling particles [18]. While these particokinetic models are appropriate for nonsoluble or poorly soluble particles, there are currently no particokinetic models for soluble particles applicable to conditions found in standard cell culture media. Mukherjee et al. (2014) [26] reported the development of a particokinetic model (ADSRM) that described diffusion, sedimentation, and dissolution in very low/high pH solutions using a Monte Carlo approach to solve for the concentration and size distribution of nanoparticles. A unique feature of the ADSRM model is its ability to model dynamic agglomeration and to incorporate detailed mechanisms of silver oxidation and citrate reduction reactions. The model was however not applied for the relevant media, nor did it consider the effects of proteins on silver nanoparticle transport and dissolution. Our density measurements combined with the ISD3 results signify that dynamic agglomeration is not important for silver nanoparticles under realistic media conditions. The one-dimensional Distorted Grid (DG) model, developed by DeLoid et al. [15], also takes into account the effects of dissolution, but the dissolution kinetics was not modeled as a particle surface area limited mechanism. Instead, the extent of dissolution was considered proportional to a reduction in agglomerated size.

Our initial efforts to predict silver nanoparticle dosimetry in our RPMI + 10% FBS in vitro test systems using ISDD, resulted in discrepancies between modeled and measured cellular doses that we hypothesized were related to the concentration, particle size, and time-dependent dissolution, and its effects on particle and ion delivery and uptake in cells. ISD3 was developed as a general modeling framework for soluble particles, adaptable to a wide range of experimental conditions, particle types, and approaches to describing sedimentation [14, 37], and dissolution. Dissolution can also be turned off, rending a model like ISDD, which can also handle spatial distributions of particles, as one might find in larger test systems, like those used in ecological testing. The modular nature of ISD3 allows the use of different boundary conditions, and models of sedimentation and dissolution. For example, instead of the instantaneous particle uptake boundary condition, other boundary conditions can also be implemented, such as, no-flux and reactive boundary conditions. Sedimentation models for agglomerates can vary depending on whether the agglomerates are permeable (as in the “particles in a box” sedimentation model [37]) or impermeable (as in ISDD [14]). Dissolution models can vary depending on the particle type, media conditions, and the kinetic processes.

### Validation of the ISD3 framework using silver Nanoparticle data

Applied to 20 and 110 nm silver nanoparticles, a central focus of the NIEHS Centers for Nanotechnology Health Implications Research (NCNHIR), ISD3 reasonably predicted the total cell content of silver, the media total silver, silver ion and particulate silver concentrations over a 24 h period. The ISD3 results were computed based on empirical models that were calibrated using the dissolution data of 20 and 110 nm silver particles, and the cell uptake data of silver ions. The population balance (PB) framework of ISD3 provided a formal mechanism for integrating the empirical models of dissolution and cell uptake with the theoretical models of particle sedimentation velocity and diffusivity. Based on the PB framework, the ISD3 model can predict changes in both the number and size of particles while the particles undergo dissolution, diffusion, and sedimentation in the liquid column, and deposition in the cells. Specifically, the model solves for the number density of particles (which is a function of the spatial location *x* and the particle diameter, *D*_*p*_) in the *x*-*D*_*p*_ parameter space, and for the total concentration of ions in the liquid media and in the cells, as a function of time. From the number density distribution, all other quantities – such as, total number, mass, concentration, size distribution, and surface area of the particles – are derived. As shown in the results, the ISD3 predictions compared well with the 24-h deposition profiles of silver in cells and with the 24-h concentration profiles of particles and ions in the liquid media.

In the current application, the silver particles were described as protein-coated primary particles with an experimentally measured effective diameter of 44 nm and 155 nm in 10% FBS for the 20 and 110 nm primary particle systems, respectively. The measured densities of the particles in FBS (1.583 g/cm^3^ for the 20 nm and 1.914 g/cm^3^ 110 nm particles) were too low to consider the particles as agglomerates but comparable to the density of proteins, suggesting the existence of a protein corona layer on the surface of silver particles (as reported in the literature [27, 28, 40]). Assuming the protein corona was protein/water, calculated densities of the 20 nm particles was ~ 1.9 g/cm^3^, very similar to the measured value. The theoretical density of the 110 nm particles was higher, ~ 4.4 g/cm^3^, but close to a factor of ~two times the measured values. Therefore, the diffusivity and sedimentation terms in the ISD3 model were evaluated based on the effective size and density of the protein-coated particles, while the dissolution term was evaluated based on the primary particle size and silver density. The thickness of the protein layer was kept fixed on all particle sizes in the simulations. It is noted that the silver particles have a gold core diameter of about 7 nm, but the dissolution was only applied to the silver, since the goal of the simulations and the experiments were only focused on silver dissolution. Therefore, in order to limit the dissolution to silver, the particles were not allowed to dissolve below a certain diameter, in the simulations. The lowest particle size was set to 10 nm in all the simulations. By the end of 24 h, particle sizes in the 20 nm system ranged from 10 to 20 nm, while the particle sizes in the 110 nm system ranged from 78 to 110 nm. Based on these size ranges, it was determined that the particle transport in the 20 and 110 nm systems was dominated by diffusion and sedimentation, respectively.

ISD3 can be applied for particles that fully dissolve during the experimental time by lowering the size threshold technically to the diameter of the ion. The size threshold need not significantly affect the delivered dose if diffusion or sedimentation (whichever is dominant) time is longer for particles near threshold sizes, but it can modify the size distribution of particles, as shown in Additional file 1: Figure S7(a) and (b) for the 20 nm particles. Difference in size distribution will modify the total surface area of the particles, which is an important determinant for the surface reactivity and toxicity of some particles. Thus, in general, the effect of setting the lowest threshold for particle size (for dissolution) on the delivered dose will depend on the initial particle size distribution, the experimental time, and the time scales for particle sedimentation, diffusion, and dissolution.

Overall, the results validate the application of the ISD3 approach for modeling dissolution effects in in vitro dosimetry studies. Further validation of the ISD3 approach to describe biosolubility can be done by generating negative results using the silver dissolution kinetic parameters for a non-soluble particle. For example, Additional file 1: Figure S8(a) shows that the % cell-associated amorphous silica particles at the end of 24 h is 30% and 32%, with and without dissolution, respectively. The difference is much larger in the liquid media (volume 0.45 ml and dish depth 0.45 cm): 59.1% and 68%, with and without dissolution, respectively (Additional file 1: Figure S8 (b)).

### Boundary conditions for particle uptake by cells

When setting the boundary conditions for the silver particle uptake by cells, it was assumed that all particles reaching the cell surface are instantaneously taken up by the cells and are no longer present in the liquid media. This assumption is reasonable because it allows for predicting the maximum possible amount of particles that can reach the cell surface. And the predicted amount of cell-associated particles can be compared with the actual deposited amount to infer how much of the particles reaching the cell surface has actually entered the cells, which in turn aids to interpret the particle uptake rates of experimentally observed dose. However, because the experiments measured total amount of cell-associated silver (includes particles and ions), the comparison between ISD3 and experiments could only be based on the total cell-associated silver rather than the individual amounts of particles and ions. Theoretically, the absorptive boundary condition for the particles (Eq. 21b) can be replaced by other boundary conditions (e.g., no-flux, reactive, or reflective), but its application depends on the system of interest. If the cellular uptake kinetics of particles is known then it can be modeled as a reactive or adsorptive term in the boundary condition, if it is biologically relevant to the system. In fact, DeLoid et al. [15] have shown that it is appropriate to use a reflective boundary condition to correctly predict the in vitro dosimetry of nanoparticles used in nanotoxicology assessments. Further studies are needed to investigate how the reflective boundary conditions affect the ISD3 predictions. For example, to understand the impact of using an absorptive compared to a purely reflective (no-flux) boundary condition, we performed simulations using a simple Robin-type of boundary condition that has one parameter (see Supporting Information). Increasing the parameter above zero causes the boundary condition change from a purely absorptive to a no-flux / purely reflective (no particles bind or enter the cells) condition. The model predicts a decrease in the cell-deposited mass of silver (Additional file 1: Figure S9), since the cell’s resistance to particle uptake increases. With appropriate boundary conditions, ISD3 can be applied to predict the deposited dose for any given particle size distribution and media height conditions, when experimental data is not available.

### Comparison between ISDD and ISD3 models

Compared to ISDD, the ISD3 model accounts for dissolution, and the population balance equation of the ISD3 model can be coupled with any models for particle dissolution and ion cell uptake kinetics. While ISDD solves for the spatial concentration of the particles, ISD3 solves for the size and spatial distribution of particle numbers, which are represented together as a particle number density (that is a function of particle location and diameter). From the number density, all other quantities, such as mass, concentration, size distribution and surface areas are derived. For the ISD3 model, the initial size distribution data can be fitted to a continuous function and then numerically discretized on to the simulation grid points when being represented by the number density. Therefore, any initial size distribution data can be represented in terms of the number density. As a result, time-dependent solutions can be obtained for all size ranges in a single run of the ISD3 simulation for a given system; which is irrespective of whether the dissolution effects are present or not. Whereas in ISDD, independent calculations have to be performed for each size class, and the results from the independent runs have to be consolidated to determine the total amount of cell-associated particles at any instant of time. Thus, like other particokinetic models (DG [15], “particles in a box” sedimentation model [37]), ISD3 can also be used to model polydispersity. If dissolution effects are not important for the system of interest, then it is recommended to use the ISDD model. If it is easier to just represent the whole initial size distribution data and run the simulation once, then the ISD3 would be a suitable approach. Like ISDD, the ISD3 model is also implemented in MATLAB. However, compared to ISDD, the ISD3 simulations take longer to run, and the simulation time increases with the particle size range in the system and the number of mesh grid points used for the particle size dimension. The current numerical integration scheme (Supporting Information) allows the integration of the sedimentation and diffusion terms (Eq. S5) for each discretized particle size value in parallel, by utilizing MATLAB’s parallelizable for-loop functionalities. Significant speed up can be achieved in the future by implementing MPI-based C/C++ versions of ISD3, following the current numerical integration scheme.

### Application of the ISD3 Particokinetic model to other particle types

The population balance equation (PBE) of the ISD3 model (Eq. 1) is a general framework for describing changes in particle populations based on the creation and loss of particles. As such, the approach we describe here for in vitro systems can be applied to any particulate system, as long as the basic assumptions of the model are met: that is, the particles are spherical in shape or can be described adequately as such; particle transport occurs only down the liquid column and is via diffusion and sedimentation (no fluid convection); and, no aggregation/agglomeration, coagulation and break-up of the particles occur during transport.

Application of ISD3 to another particle system would involve inputting parameters appropriate for the particles and model system (Table 1). Media height, media viscosity, density, and particle or agglomerate size and density are the primary parameters necessary for model application. These are commonly available or readily measured parameters. The ISD3 code is modular, allowing use of the existing dissolution model, which may or may not be appropriate for other particles, or an alternate developed by the user. Particle uptake by cells, similarly, can be described as instantaneous, as we have here, or revised to reflect the kinetics of the new system. The ion uptake model can also be revised, and the remaining equations in the code remain the same. The empirical models for dissolution and cell ion uptake would need to be refitted to particle specific data, and the initial size distribution would be determined by DLS and input into the model. The empirical models developed for the dissolution of silver nanoparticles and for the cell uptake of silver ions may be applicable to particles of other material types, if they capture all the physical processes relevant to the other particle systems. The parameters of silver particles and ions may not be transferrable to particles of other material types; and therefore, the kinetic models will need to be re-fitted using the experimental data that is unique to each material.

### Understanding sources of error in the use of ISD3

Discrepancies can arise between the ISD3 predicted values and experimental values due to factors that may not be accounted for by the model or controlled for in the experiments. For example, in the current simulations with silver nanoparticles, the values for the amount of cell-associated silver did not match the experimental (average) values at 24 h, but the time-deposition profiles are similar given the experimental error bars. Except for one system (12.5 μg/mL and 20 nm system), for all other systems, the predicted value at 24 h was higher than the experimental value. Such over-predictions by the ISD3 model can be attributed to the instantaneous boundary condition used for the uptake of the particles in the cells. Apparently, not all particles reaching the cell surface are taken up by the cells, which is a valuable inference made by the model, also reported by DeLoid et al. [15]. It is also possible for the measured values to be lower than what is actually present in the system. For example, particles associated with the cell surface may get lost during washing and separation or the cells may be shutting down intake after some time, which can lower the measured values. Whatever the case might be, the system is too complex to determine the actual source for the discrepancies. In the case of 12.5 μg/mL and 20 nm system, ISD3 underestimated the amount of deposited silver at the end of 24 h. From the ISD3 point of view, such a discrepancy can arise if the actual initial spatial distribution of the particles was not uniform along the liquid column. All ISD3 simulations started with a uniform spatial distribution of the particles. As we showed, diffusivity dominates the transport of particles in the 20 nm system. Therefore, it will take more time for these particles to reach the cell surface from a height by diffusion than by sedimentation. But if there were relatively more particles close to the cell surface, such that they would have diffused to the cell surface within 24 h, then the ISD3 predictions would have been higher. Nevertheless, the discrepancies are small given the large error bars observed for the measured values. Hence, overall, the ISD3 predictions compared well with experiments, and confirmed the important role of dissolution effects in estimating the amount of cell-associated silver in the cells and the concentration of ions in the liquid column.

### Use of ISD3 for modeling intracellular concentrations of ions and particles

Both extracellular (e.g. sedimentation, dissolution) and internal processes (intracellular trafficking, dissolution, export), contribute to cellular dosimetry in in vitro systems. Like ISDD, in developing ISD3, the focus remained of using system characteristics and particle properties to describe delivery of particles and ions to cells, and not cellular uptake and processing, which would be expected to be highly cell-type specific. A very promising approach to dosimetry modeling of particles and their soluble ions would be combining ISD3 with models of intracellular fate and transport of particles and ions. This approach has the advantage of using one model, ISD3, to control for the influence of the rate and extent of “delivery” of ions and particles to cells on measurements and modeling of intracellular trafficking. De-convolution of these two contributors is necessary for studying intracellular dosimetry.

## Conclusions

Sustained development and improvement of experimental and computational methods characterizing nanomaterial cellular exposures in vitro and in vivo continues to improve the basis for both hazard ranking and risk assessment of nanomaterials. ISD3 is a valuable extension of ISDD and other models to describe the influence of dissolution on the cellular dosimetry of soluble nanoparticles such as silver. With the flexibility to replace descriptions of dissolution, agglomerate sedimentation and boundary conditions with those appropriate for particles other than silver, ISD3 can be adapted to new applications. Combining experiments and modeling, we were able to quantify the influence of proteins on particle solubility, determine the relative amounts of silver ions and particles in exposed cells, and demonstrate the influence of particle size changes resulting from dissolution on particle delivery to cells in culture.

## Additional file

Additional file 1: Figure S1. Model predictions of total dissolved silver for different initial concentrations of free and bound ions and 12.5 μg/mL particle concentration in RPMI + 1% (a), 10% (b) and 30% (c) FBS. **Figure S2.** Collinearity of parameters (in log10 scale) as a function of the number of parameters selected. **Figure S3.** Sensitivity range of model predictions for total dissolved silver to each parameter of the model, while keeping the other parameters fixed at their fitted values. **Figure S4.** Sensitivity range of model predictions for total dissolved silver concentration for 12.5 μg/ml particle concentration in RPMI + 1% (a), 10% (b) and 30% (c) FBS, to 10% change in all parameters. **Figure S5**. Number of particles in the liquid media as a function particle diameter (*Dp*) at selected time points from 0.024 to 24 h, starting with: a) 12.5 μg/mL concentration of 20 nm particles; and, b) 9.15 μg/mL concentration of 110 nm particles. **Figure S6.** Péclet number as a function particle diameter (*Dp*) based on particle densities, 1.583 g/cm3 (20 nm system) (a) and 1.914 g/cm3 (110 nm system) (b). **Figure S7.** Effect of changing the lower limit for the particle size from 10 nm to 2 nm (due to dissolution) in the 20 nm system with an initial concentration of 1 μg/mL: (a) Particle number density versus particle diameter. **Figure S8.** Negative results showing the effect of dissolution on the percentage dose of silica in cells (a) and in the liquid media (b), using silver dissolution kinetics. **Figure S9.** Effect of increasing the cell’s resistance to particle uptake on deposited mass of silver for 20 nm particles, by varying the parameter *K* from 0 to 0.05. (PDF 1772 kb)

## Abbreviations

ADSRM: Agglomeration-diffusion-sedimentation-reaction
AgNP: Silver nanoparticles
DG: Distorted Grid
DLS: Dynamic Light Scattering
FBS: Fetal bovine serum
ISD3: In vitro sedimentation, diffusion, dissolution, and dosimetry
ISDD: In vitro sedimentation, diffusion, and dosimetry
PBE: Population balance equation
TEM: Transmission Electron Microscopy
VCM: Volumetric centrifugation method
