# Supplementary material for: ISD3: a particokinetic model for predicting the combined effects of particle sedimentation, diffusion and dissolution on cellular dosimetry for in vitro systems
Source: Part Fibre Toxicol. 2018 Jan 25;15:6. doi: 10.1186/s12989-018-0243-7 (PMC5784555; doi:10.1186/s12989-018-0243-7)
Supplement: Additional file 1: Figure S1. — Model predictions of total dissolved silver for different initial concentrations of free and bound ions and 12.5 μg/mL particle concentration in RPMI + 1% (a), 10% (b) and 30% (c) FBS. Figure S2. Collinearity of parameters (in log10 scale) as a function of the number of parameters selected. Figure S3. Sensitivity range of model predictions for total dissolved silver to each parameter of the model, while keeping the other parameters fixed at their fitted values. Figure S4. Sensitivity range of model predictions for total dissolved silver concentration for 12.5 μg/ml particle concentration in RPMI + 1% (a), 10% (b) and 30% (c) FBS, to 10% change in all parameters. Figure S5. Number of particles in the liquid media as a function particle diameter (Dp) at selected time points from 0.024 to 24 h, starting with: a) 12.5 μg/mL concentration of 20 nm particles; and, b) 9.15 μg/mL concentration of 110 nm particles. Figure S6. Péclet number as a function particle diameter (Dp) based on particle densities, 1.583 g/cm3 (20 nm system) (a) and 1.914 g/cm3 (110 nm system) (b). Figure S7. Effect of changing the lower limit for the particle size from 10 nm to 2 nm (due to dissolution) in the 20 nm system with an initial concentration of 1 μg/mL: (a) Particle number density versus particle diameter. Figure S8. Negative results showing the effect of dissolution on the percentage dose of silica in cells (a) and in the liquid media (b), using silver dissolution kinetics. Figure S9. Effect of increasing the cell’s resistance to particle uptake on deposited mass of silver for 20 nm particles, by varying the parameter K from 0 to 0.05. (PDF 1772 kb) [file 12989_2018_243_MOESM1_ESM.pdf]

ISD3: A Particokinetic Model for Predicting the Combined Effects of Particle Sedimentation, Diffusion and Dissolution on Cellular Dosimetry for In Vitro Systems

Dennis G. Thomas[1,*], Jordan N. Smith[2], Brian D. Thrall[2], Donald R. Baer[3], Hadley Jolley[2], Prabhakaran Munusamy[3], Vamsi Kodali[2], Philip Demokritou[4], Joel Cohen[4], Justin G. Teeguarden[2,5,*]

[1]Computational Biology, Biological Sciences Division, Pacific Northwest National Laboratory, Richland, WA 99352

[2]Health Effects and Exposure Science, Biological Sciences Division, Pacific Northwest National Laboratory, Richland, WA 99352

[3]Interfacial Sciences and Simulation, Environmental Molecular Sciences Division, Pacific Northwest National Laboratory, Richland, WA 99352

[4]Center for Nanotechnology and Nanotoxicology, Department of Environmental Health, Harvard University T. H. Chan School of Public Health, Boston, MA 02115

[5]Department of Environmental and Molecular Toxicology, Oregon State University, Corvallis, OR 93771

*Corresponding author(s)
902 Battelle Blvd
Richland, WA 99352
dennis.thomas@pnnl.gov, 509-375-6793
jt@pnnl.gov, 509-375-6981

Supporting Information

Effect of initial percentage of free and bound ion concentrations in the silver dissolution model

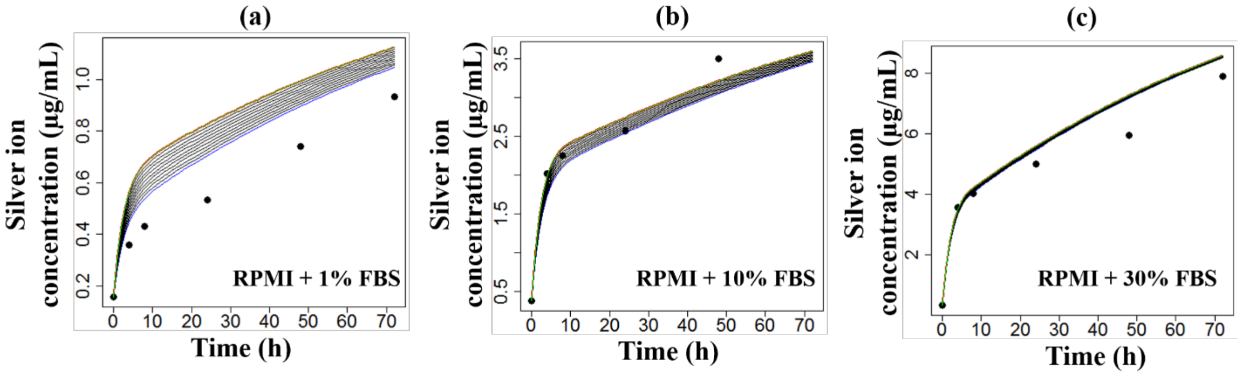

Figure S1. Model predictions of total dissolved silver for different initial concentrations of free and bound ions and 12.5 μg/mL particle concentration in RPMI + 1% (a), 10% (b) and 30% (c) FBS. Initial concentration of free ions ranged from 0 to 100% of the observed total dissolved silver at time 0, in 10% increments. Model predictions with initial 0%, 85%, and 100% free ions (equivalent to 100%, 15%, and 0% bound ions) are indicated by blue, red, and green lines, respectively. Points represent experimental data.

Parameter identifiability analysis of the dissolution model

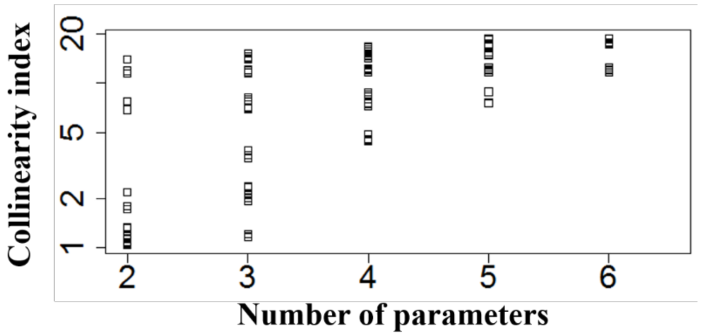

Figure S2. Collinearity of parameters (in $\log_{10}$ scale) as a function of the number of parameters selected. Only values less than 20 are plotted. The larger the collinearity value, the less identifiable the parameter based on the data.

Parameter sensitivity analysis of the dissolution model

Sensitivity to individual parameters

Parameter | **RPMI + 1% FBS** | **RPMI + 10% FBS** | **RPMI + 30% FBS**

$C_{sat}^{diss,f}$

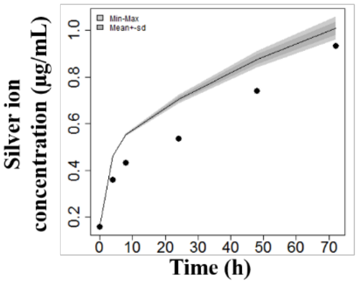

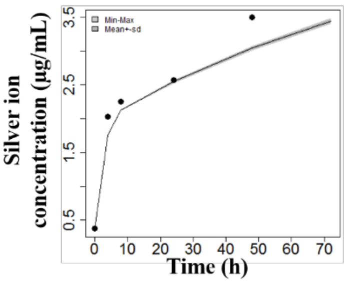

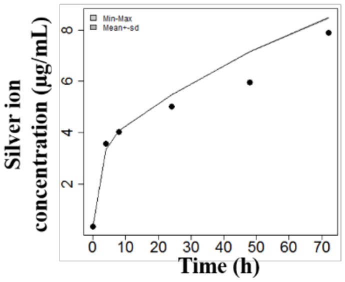

k_f

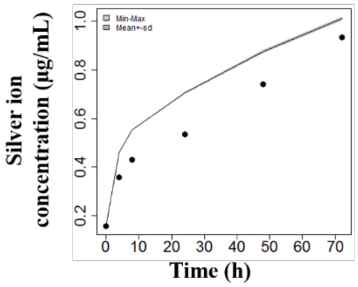

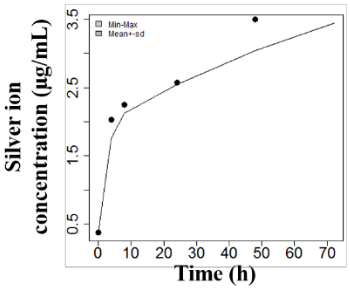

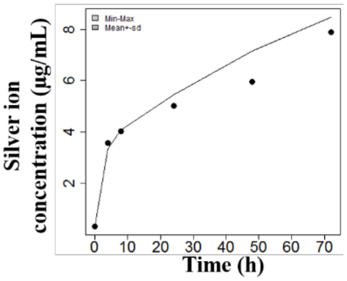

Parameter | **RPMI + 1% FBS** | **RPMI + 10% FBS** | **RPMI + 30% FBS**

k_{f2p}

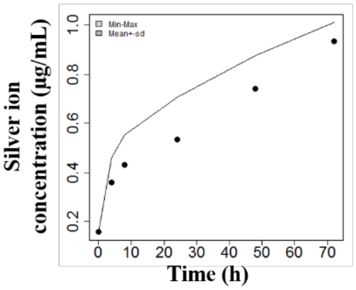

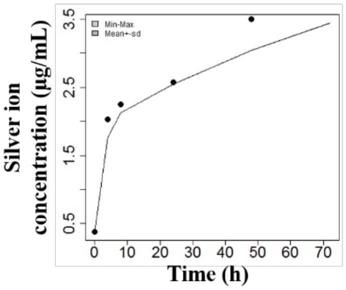

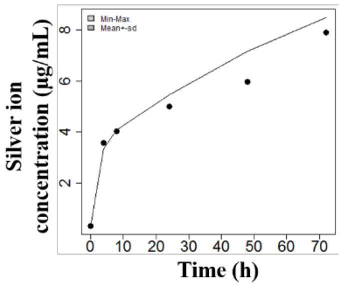

k_p

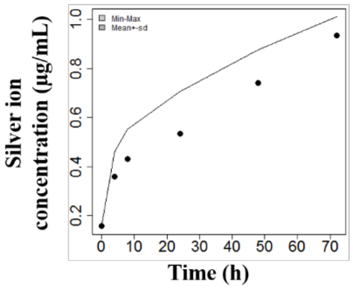

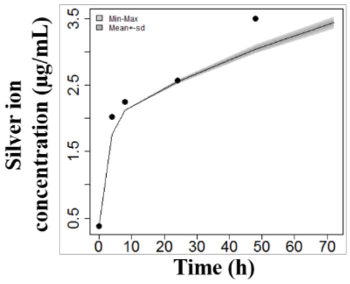

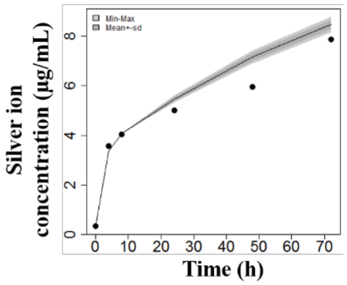

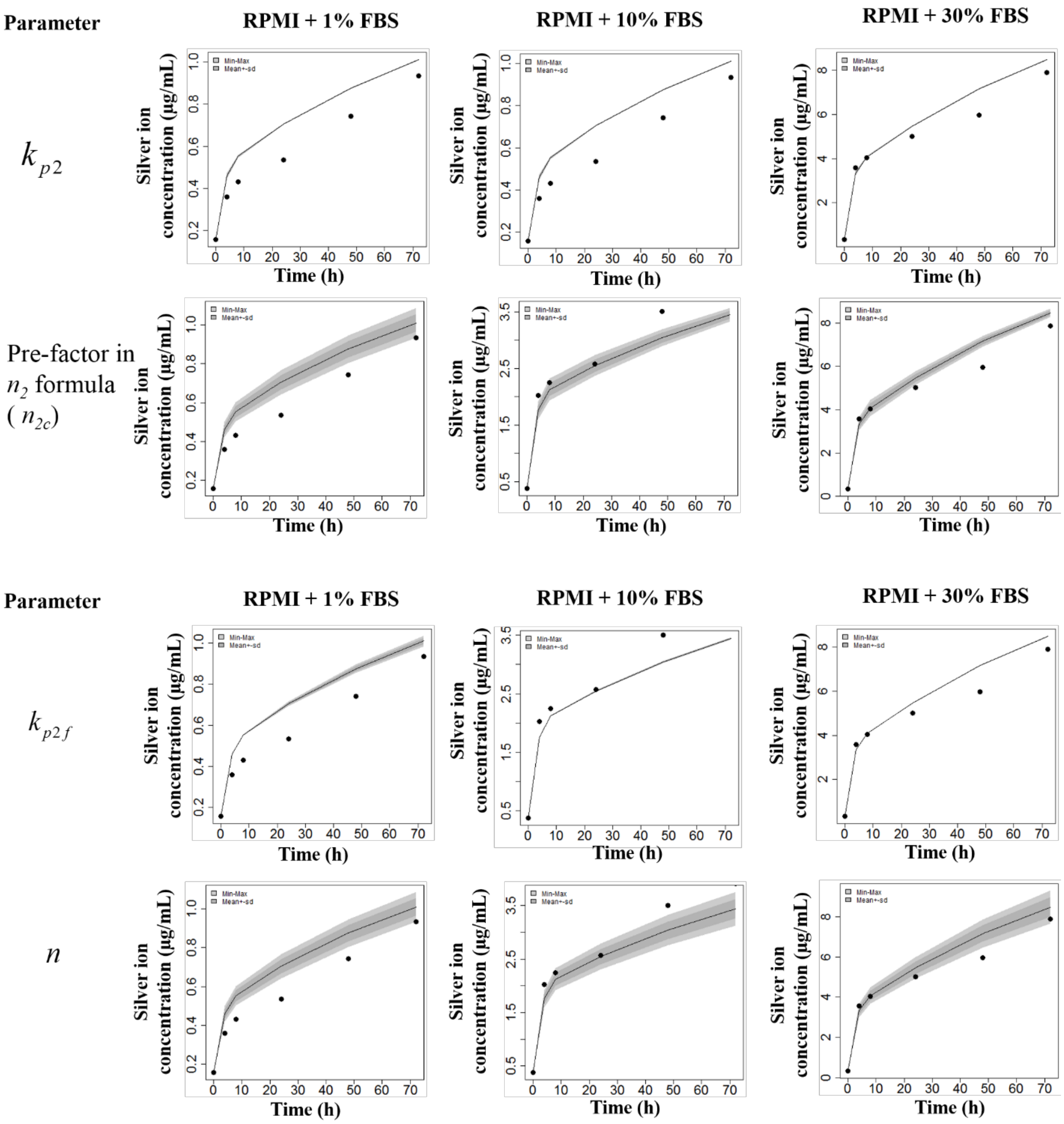

Figure S3. Sensitivity range of model predictions for total dissolved silver to each parameter of the model, while keeping the other parameters fixed at their fitted values. Results shown for the dissolution data (12.5 µg/mL particle concentration in RPMI + 1%, 10% and 30% FBS) used for fitting the parameters. Individual parameter values ranged within 10% of their fitted values. The points represent experimental data. Results were obtained using the *sensRange* function from the FME package in R.

Sensitivity to all parameters

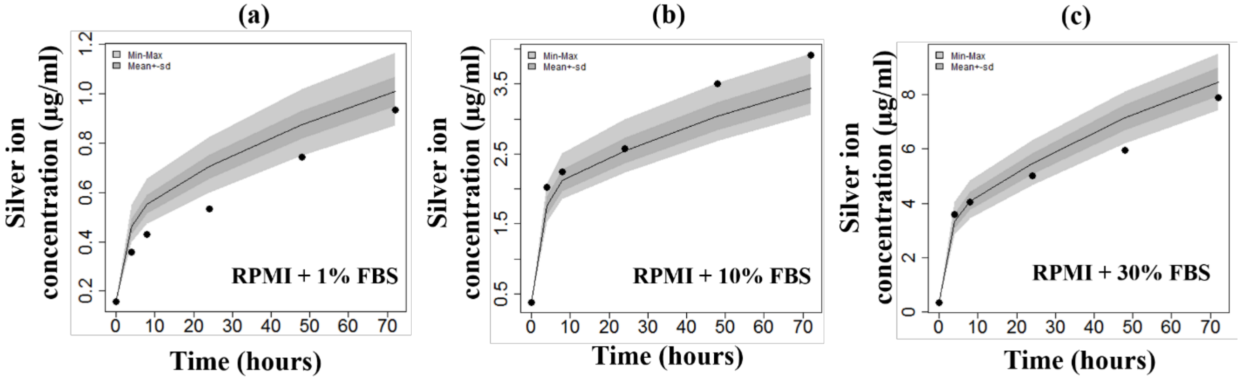

Figure S4. Sensitivity range of model predictions for total dissolved silver concentration for 12.5 µg/ml particle concentration in RPMI + 1% (a), 10% (b) and 30% (c) FBS, to 10% change in all parameters. Results were obtained using the *sensRange* function from the FME package in R. The parameters were sampled using the latin hypercube sampling algorithm.

Change in particle number and size in the liquid media

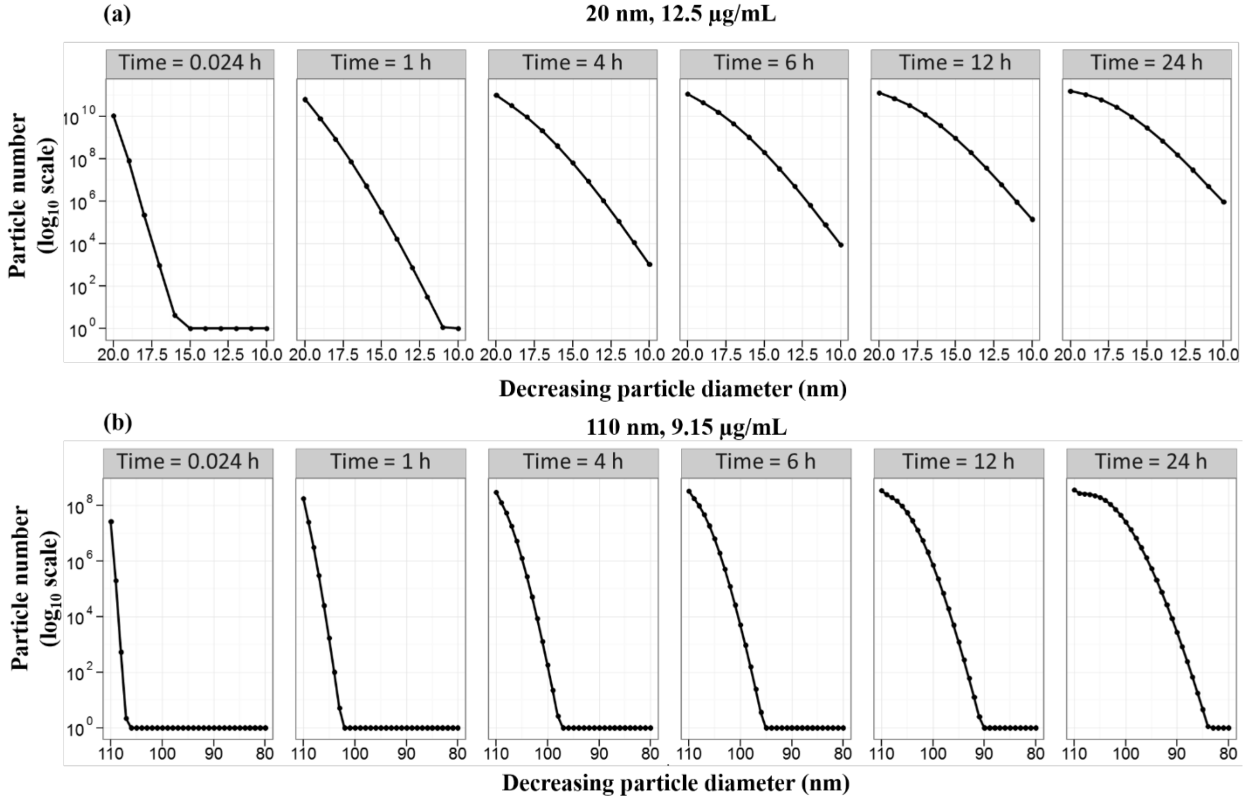

Figure S5. Number of particles in the liquid media as a function particle diameter (D_p) at selected time points from 0.024 to 24 hours, starting with: a) 12.5 µg/mL concentration of 20 nm particles; and, b) 9.15 µg/mL concentration of 110 nm particles.

Dependence of Péclet number on particle diameter

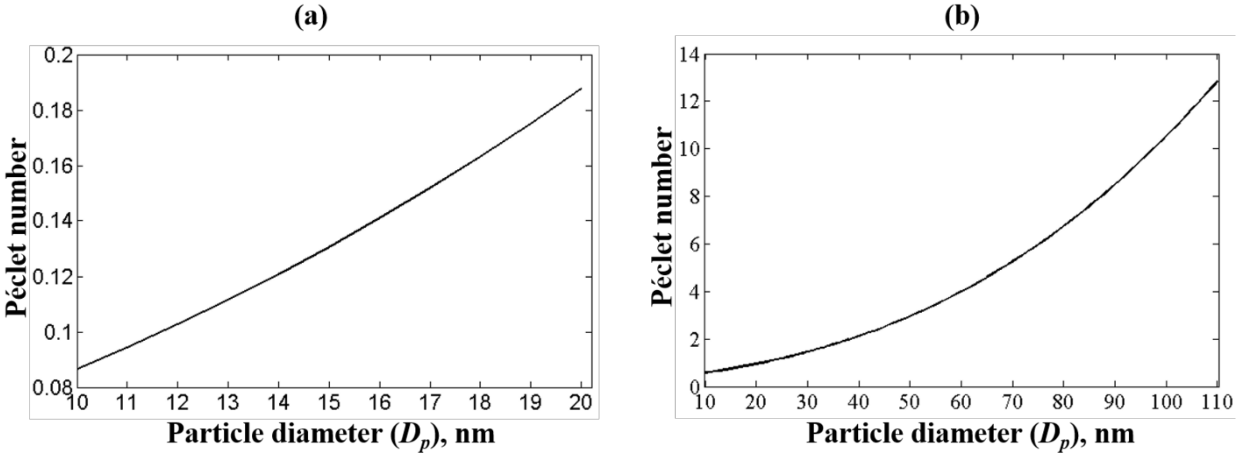

Figure S6. Péclet number as a function particle diameter (D_p) based on particle densities, 1.583 g/cm^3 (20 nm system) (a) and 1.914 g/cm^3 (110 nm system) (b). Note the diameter used for calculating the Péclet number is $D_p + 2\Delta R_c$, where R_c = 12 nm for the 20 nm system and 22.5 nm for 110 nm system.

Effect of lowering the nanoparticle size threshold for dissolution

For particles that fully dissolve during the experimental time, the lowest size threshold for dissolution should be set to a non-zero value, technically the diameter of the ion. Lowering the size threshold need not affect the delivered dose if diffusion or sedimentation (whichever is dominant) time is longer for particles near threshold sizes, but it can modify the size distribution of particles, as shown in Figures S7(a) and S7(b) for the 20 nm particles. Based on the size distribution, the total surface area of the particles will change, which can impact the surface reactivity and toxicity of particles.

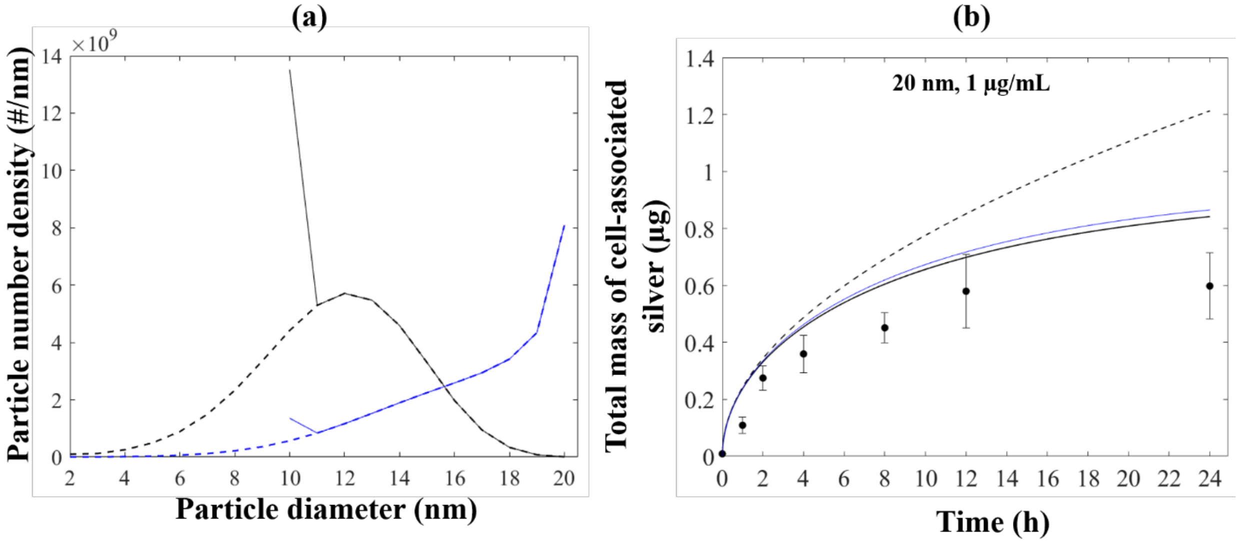

Figure S7. Effect of changing the lower limit for the particle size from 10 nm to 2 nm (due to dissolution) in the 20 nm system with an initial concentration of 1 µg/mL: (a) Particle number density versus particle diameter. Black and blue lines represent the particle number density in the liquid media and in the cells, respectively. Solid and dashed lines are the results obtained when the particle size limit was 10 nm and 2 nm, respectively. (b) Total mass of cell-associated silver versus time. This figure is similar to figure 5, but with an additional line (solid blue) showing the result when the lower limit was set to 2 nm.

ISD3 simulations for a non-soluble particle (35 nm amorphous silica) with and without dissolution

Simulations were performed with 35-nm silica particles to obtain negative data for further proving the model's ability to account for biosolubility. The initial particle concentration was 37 µg/mL and uniformly distributed in FBS. Media volume was 0.45 mL and dish depth was 0.45 cm. The silver dissolution kinetic parameters were used to generate the negative data. The percentage dose of silica in the cells and in the liquid media is shown in Figure S8. The % cell-associated silica particle at the end of 24 hours was found to be 30% and 32%, with and without dissolution, respectively. The difference is much larger in the liquid media: 59.1 % and 68%, with and without dissolution, respectively. The particle surface area and size distribution will change if particles dissolve, which can modify the surface reactivity and toxicity of

nanoparticles. Thus, ISD3 can account for bio-solubility as long as experimentally validated models are used for dissolution kinetics.

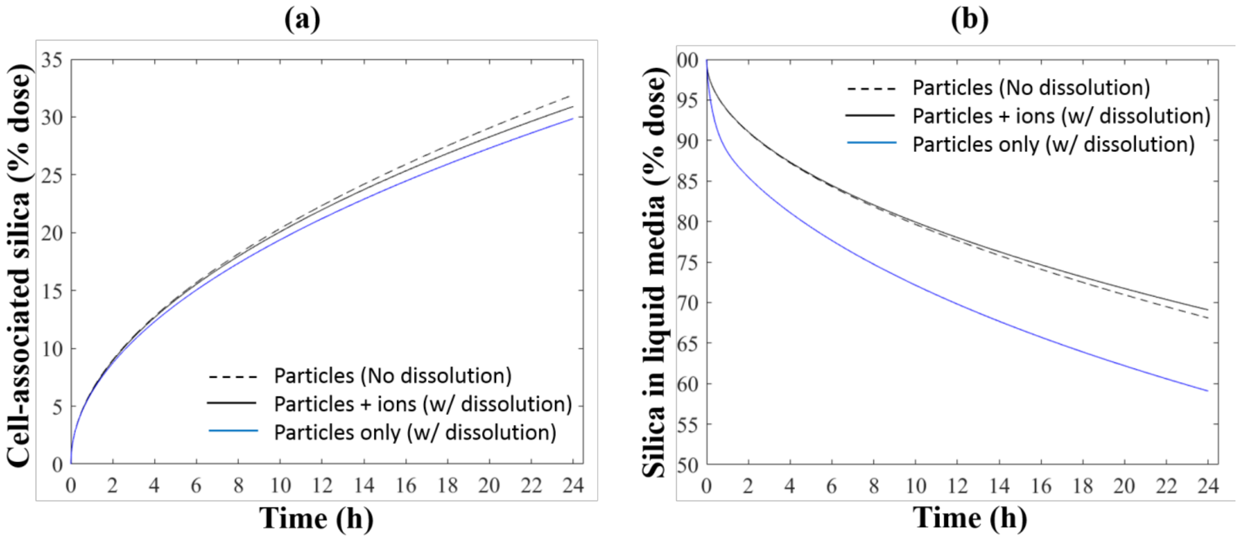

Figure S8. Negative results showing the effect of dissolution on the percentage dose of silica in cells (a) and in the liquid media (b), using silver dissolution kinetics.

Effect of no-flux and absorptive boundary conditions in ISD3

To understand the impact of using a purely absorptive boundary condition (Equation 21b), we performed simulations using a simple Robin-type of boundary condition that has one parameter, *K*, as shown below:

$$N(D_p;x,t)+K\frac{\partial N(D_p;x,t)}{\partial x}=0$$

Increasing *K* continuously from zero will make the boundary condition change from a purely absorptive to a no-flux (no particles bind or enter the cells) condition. Figure S9 shows that the total mass of silver deposited in the cells will decrease when *K* is increased from 0 to 0.05, which is expected because the cell's resistance to particle uptake increases with increasing *K*.

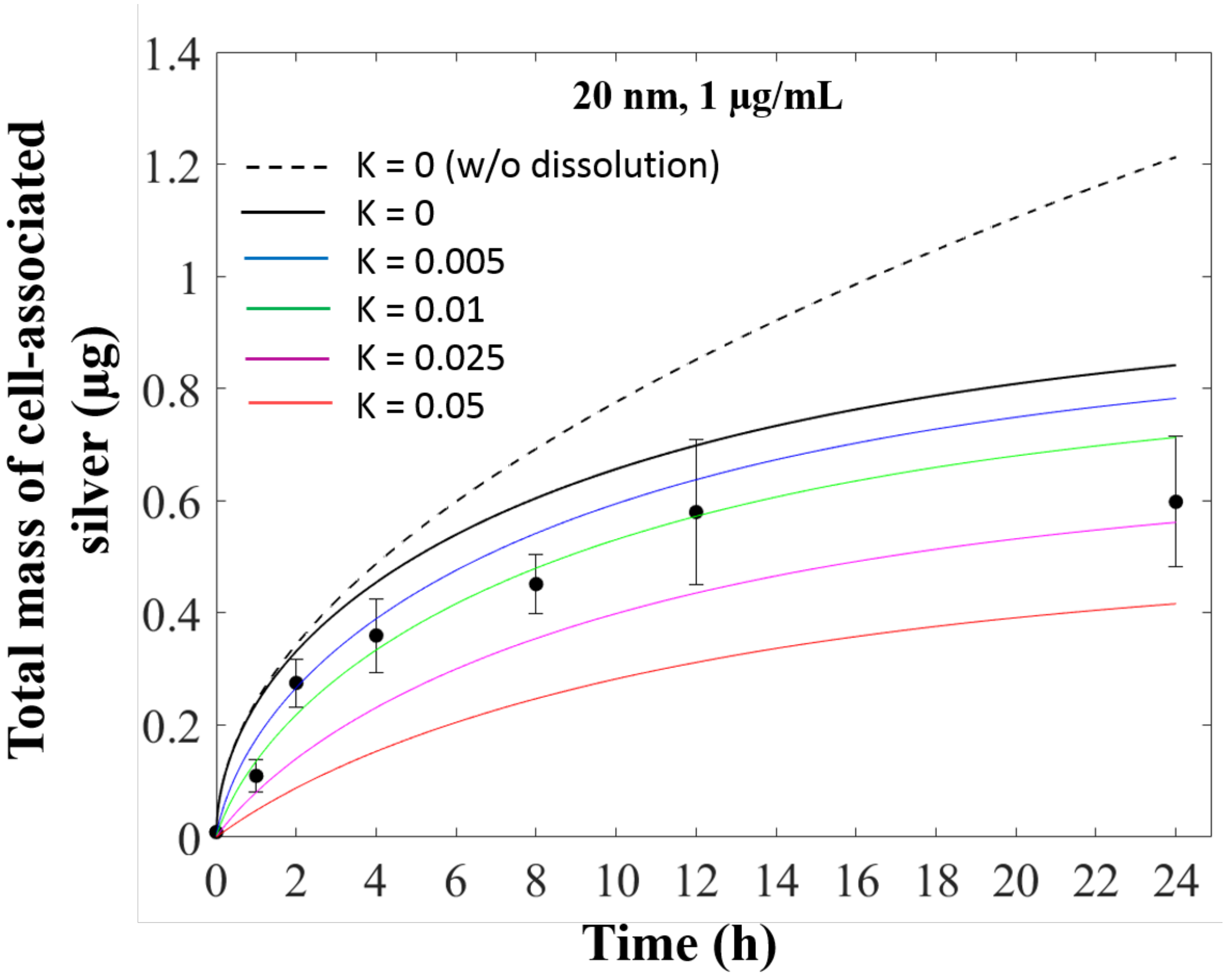

Figure S9. Effect of increasing the cell's resistance to particle uptake on deposited mass of silver for 20 nm particles, by varying the parameter K from 0 to 0.05.

Time integration of the ISD3 equations

Discretization of the time, particle diameter, and spatial domains

The time domain, $0 \le t \le t_{\max}$, is discretized as

$$t_i = i \cdot \Delta t; \qquad i = 0,1,...,M_t\,; M_t = \frac{t_{\max}}{\Delta t} \qquad \text{S-1}$$

The particle size (internal physical coordinate) domain, $D_{p,\min} \le D_p \le D_{p,\max}$, is discretized as

$$D_{p;j} = D_{p,\min} + j \cdot \Delta D_p; \qquad j = 0, 1, ..., M_{Dp}; \qquad M_{Dp} = \frac{(D_{p,\max} - D_{p,\min})}{\Delta D_p} \qquad \text{S-2}$$

The spatial (external physical coordinate) domain, $0 \le x \le L$, is discretized as

$$x_k = k \cdot \Delta x; \qquad k = 0, 1, ..., M_x; \qquad M_x = \frac{L}{\Delta x} \qquad \text{S-3}$$

Integration of the ISD3 equations

We use a fully implicit, multi-step time integration procedure to solve the equations of the ISD3 model (Equations 12, 13, and 20) along with the initial (Equations 24, 25, 27 and 28) and boundary (Equations 21a and 21b) conditions. At each time step, t_i ($i > 0$), the solutions for the number density ($N(D_p; x, t_i) \equiv N_i$), dissolved (ion) concentration (C_i^{diss}), and surface area ($Area_i$) of particles are implicitly solved through an iterative procedure until a specified convergence criterion is met. Particularly, at any given iteration, *q*, the solution for N_{i+1} at time t_{i+1} is updated in two steps after splitting Equation 20 into two equations:

$$\frac{\partial N_{i+1/2,q}}{\partial t} = \frac{\partial}{\partial D_p}\left(\frac{2}{\rho_p} a\left(k_f\left(C_{sat}^{diss,f} - C_{i+1;q}^{diss,f}\right) + k_p\left(n \cdot P_0 - C_{i+1;q}^{diss;p}\right) + k_{p2}\left(n_2 \cdot P_0 - C_{i+1;q}^{diss,p}\right)\right) N_{i+1/2;q}\right) \text{S-4}$$

and,

$$\frac{\partial N_{i+1;q}}{\partial t} = D_{diff}(D_p) \frac{\partial^2 N_{i+1;q}}{\partial x^2} - V_t(D_p) \frac{\partial N_{i+1;q}}{\partial x} \qquad \text{S-5}$$

In the first step, Equation S-4 is integrated using N_i as the initial solution to obtain an intermediate solution, $N_{i+1/2;q}$; which, serves as the initial condition for integrating Equation S-5 to obtain the solution, $N_{i+1;q}$.

Specifically, Equation S-4 is time-integrated using the Adam-Bashforth (A-B) method as follows:

For $q=1$ and $i=0$,

$$N_{i+1/2,q} = N_i + \Delta t \frac{\partial}{\partial D_p}\left(\frac{2}{\rho_p} a\left(k_f\left(C_{sat}^{diss,f} - C_i^{diss,f}\right) + k_p\left(n \cdot P_0 - C_i^{diss;p}\right) + k_{p2}\left(n_2 \cdot P_0 - C_i^{diss,p}\right)\right) N_i \right)$$

(explicit 1st order A-B (forward Euler)) S-6

For $q=1$, and $i \geq 1$

$$N_{i+1/2,q} = N_i + \frac{\Delta t}{2}\left[\begin{array}{l} 3\frac{\partial}{\partial D_p}\left(\frac{2}{\rho_p} a\left(k_f\left(C_{sat}^{diss,f} - C_i^{diss,f}\right) + k_p\left(n \cdot P_0 - C_i^{diss;p}\right) + k_{p2}\left(n_2 \cdot P_0 - C_i^{diss,p}\right)\right) N_i \right) \\ -\frac{\partial}{\partial D_p}\left(\frac{2}{\rho_p} a\left(k_f\left(C_{sat}^{diss,f} - C_{i-1}^{diss,f}\right) + k_p\left(n \cdot P_0 - C_{i-1}^{diss;p}\right) + k_{p2}\left(n_2 \cdot P_0 - C_{i-1}^{diss,p}\right)\right) N_{i-1} \right) \end{array} \right]$$

(explicit 2nd order A-B). S-7

For $q \geq 2$,

$$N_{i+1/2,q} = N_i + \frac{\Delta t}{2}\left[\begin{array}{l} \frac{\partial}{\partial D_p}\left(\frac{2}{\rho_p} a\left(k_f\left(C_{sat}^{diss,f} - C_{i+1;q-1}^{diss,f}\right) + k_p\left(n \cdot P_0 - C_{i+1;q-1}^{diss;p}\right) + k_{p2}\left(n_2 \cdot P_0 - C_{i+1;q-1}^{diss,p}\right)\right) N_{i+1/2;q-1}\right) \\ + \frac{\partial}{\partial D_p}\left(\frac{2}{\rho_p} a\left(k_f\left(C_{sat}^{diss,f} - C_i^{diss,f}\right) + k_p\left(n \cdot P_0 - C_i^{diss;p}\right) + k_{p2}\left(n_2 \cdot P_0 - C_i^{diss,p}\right)\right) N_i\right) \end{array}\right]$$

(implicit 2nd order A-B (Crank-Nicholson)) S-8

The solution of $N\left(D_p = D_{p.j}; x, t\right)$ depends only on the solution downstream of D_p ($D_p > D_{p.j}$). Therefore, the derivative of the particle number density with respect to diameter at a value, $D_p = D_{p,j}$, is evaluated using the (1st order) upwind finite differencing scheme, as shown below:

$$\frac{\partial N}{\partial D_p}\left(D_p = D_{p.j}; x, t_i\right) = \frac{N\left(D_p = D_{p.j+1}; x, t_i\right) - N\left(D_p = D_{p.j}; x, t_i\right)}{\Delta D_p}. \qquad \text{S-9}$$

Equation S-5 is integrated using the ‘pdepe’ function in Matlab, where the implemented boundary conditions are

$$D_{diff}\,(D_p)\frac{\partial N_{i+1;q}}{\partial x} - V_t\,(D_p)\; N_{i+1;q} = 0 \;\text{ at } x = 0 \text{ (top surface),} \qquad \text{S-10}$$

and,

$$N_{i+1;q} = 0 \qquad \text{at } x = L \text{ (bottom surface),} \qquad \text{S-11}$$

Calculation of dissolved (unbound ion) mass concentration in the media

The solution, $C_{i+1;q}^{diss,f}$, is obtained by integrating Equation 12 over a time step of Δt, using Matlab’s ‘ode45’ solver for ordinary differential equations,

$$\frac{dC_{i+1;q}^{diss,f}}{dt} = \frac{k_f \ Area_i}{V}\left(C_{sat}^{diss,f} - C_{i+1;q}^{diss,f}\right) - k_{f2p}C_{i+1;q}^{diss,f}\left(n \cdot P_0 - C_{i+1;q}^{diss,p}\right)$$

$$+ k_{p2f}C_{i+1;q}^{diss,p}\left(C_{sat}^{diss,f} - C_{;i+1;q}^{diss,f}\right) - \frac{D_{12}SA_2}{V}\frac{\left(C_{i+1;q}^{diss,f} - \frac{C_{i+1;q}^{diss,cell}V/V_{cell}}{PC_{21}}\right)}{Dis_2}$$

for $q = 1$ S-12

or

$$\frac{dC_{i+1;q}^{diss,f}}{dt} = \frac{k_f \ Area_{i+1;q-1}}{V}\left(C_{sat}^{diss,f} - C_{i+1;q}^{diss,f}\right) - k_{f2p}C_{i+1;q}^{diss,f}\left(n \cdot P_0 - C_{i+1;q}^{diss,p}\right)$$

$$+ k_{p2f}C_{i+1;q}^{diss,p}\left(C_{sat}^{diss,f} - C_{i+1;q}^{diss,f}\right) - \frac{D_{12}SA_2}{V}\frac{\left(C_{i+1;q}^{diss,f} - \frac{C_{i+1;q}^{diss,cell}V/V_{cell}}{PC_{21}}\right)}{Dis_2}$$

for $q \geq 2$ S-13

Similarly, $C_{i+1;q}^{diss,p}$, is obtained by integrating Equation 13 ,

$$\frac{dC_{i+1;q}^{diss,p}}{dt} = \frac{k_p \ Area_i}{V}\left(n \cdot P_0 - C_{i+1;q}^{diss,p}\right) + \frac{k_{p2} \ Area_i}{V}\left(n_2 \cdot P_0 - C_{i+1;q}^{diss,p}\right)$$

$$+ k_{f2p}C_{i+1;q}^{diss,f}\left(n \cdot P_0 - C_{i+1;q}^{diss,p}\right) - k_{p2f}C_{i+1;q}^{diss,p}\left(C_{sat}^{diss,f} - C_{i+1;q}^{diss,f}\right)$$

for $q = 1$, S-15

or

$$\frac{dC_{i+1;q}^{diss,p}}{dt} = \frac{k_p \ Area_{i+1;q-1}}{V}\left(n \cdot P_0 - C_{i+1;q}^{diss,p}\right) + \frac{k_{p2} \ Area_i}{V}\left(n_2 \cdot P_0 - C_{i+1;q}^{diss,p}\right)$$
$$+ k_{f2p} C_{i+1;q}^{diss,f}\left(n \cdot P_0 - C_{i+1;q}^{diss,p}\right) - k_{p2f} C_{i+1;q}^{diss,p}\left(C_{sat}^{diss,f} - C_{i+1;q}^{diss,f}\right)$$

for $q \geq 2$. S-16

Equations S-15 and S-16 are numerically solved using $C_i^{diss,f}$ and $C_i^{diss,p}$ as the initial conditions when q = 1, and $C_{i+1;q-1}^{diss,f}$ and $C_{i+1;q-1}^{diss,p}$ when $q \geq 2$. The difference between Equations S-13 and S-14, and between Equations S-15 and S-16, is in the numerical solution used for the total particle surface area: $Area_i$ in when $q = 1$, and $Area_{i+1,q-1}$ when $q \geq 2$.

Convergence criterion

The convergence criterion is defined such that the absolute value of the difference in the total particle surface area between the (q+1)th and (q)th iteration should be less than or equal to a tolerance value, $\varepsilon = 1$ nm^2; i.e.,

$$\left|Area_{i+1,q} - Area_{i+1,q-1}\right| \leq \varepsilon \qquad \text{S-17}$$

where, the total particle surface area, in the (i+1)th time integration step, is calculated as

$$Area_{i+1;q} = \iint N_i \, \pi D_p^2 \, dD_p \, dx \quad \text{for} \quad q = 1. \qquad \text{S-18}$$

$$Area_{i+1;q} = \iint N_{i+1;q} \pi D_p^2 \, dD_p \, dx \quad \text{for} \quad q \geq 2. \qquad \text{S-19}$$

Additionally, mass is conserved at each time step by controlling the absolute error in the total concentration to a value $\leq 10^{-5}$ μg/mL.
